# In Vivo CRISPR Screen Identifies RPS19 as an Immunotherapy Target in Hepatocellular Carcinoma

**DOI:** 10.1002/advs.78430

**Published:** 2026-10-25

**Authors:** Lei He, Duo Yu, Fei Xie, Kaisheng Hou, Zhaozhao Li, Meifang Dai, Jingyi Yun, Haiyan Cao, Yang Xiao, Yueyuan Qiu, Gege Shi, Wangqian Zhang, Shuning Wang, Qiang Hao, Jieyu Zhang, Zhaowei Wang, Yingqi Zhang, Kuo Zhang, Jintao Gu, Haichen Du, Meng Li

**Affiliations:** ^1^ State Key Laboratory of Holistic Integrative Management of Gastrointestinal Cancers Department of Biopharmaceutics School of Pharmacy Fourth Military Medical University Xi'an China; ^2^ Department of Neurosurgery General Hospital of Central Theater Command Wuhan China; ^3^ Department of Neurosurgery Tangdu Hospital Fourth Military Medical University Xi'an China; ^4^ Department of Experiment Surgery Xijing Hospital Fourth Military Medical University Xi'an China; ^5^ Department of Oncology 940th Hospital Joint Logistic Support Force Lanzhou China

**Keywords:** CRISPR screens, gene editing, hepatocellular carcinoma, RPS19, tumor immunotherapy

## Abstract

Despite the remarkable clinical success of immunotherapy, its efficacy against hepatocellular carcinoma (HCC) remains severely limited, highlighting an urgent need for novel targets and strategies to overcome immune evasion. Here, we aimed to identify key regulators of HCC immunity via unbiased functional screening and develop a targeted therapy to remodel the immunosuppressive tumor microenvironment (TME). We performed an in vivo CRISPR‐knockout screen using a 4500‐gene sgRNA library in syngeneic HCC mouse models. Single‐cell RNA sequencing of human HCC tissues and in vivo macrophage‐depletion assays were utilized to dissect cellular populations and validate underlying mechanisms. We identified ribosomal protein S19 (RPS19) as a central mediator of HCC immune evasion, whose deletion in HCC cells mitigates the immunosuppressive TME. Mechanistically, scRNA‐seq pinpointed tumor‐associated macrophages as the key effector subset, whereby complement C5a receptor 1 (C5aR1) transduces RPS19 signals to trigger macrophage‐dependent immune suppression. We further constructed SP94‐peptide‐modified disulfide‐bond‐containing nanoparticles (SNP_SS_) for targeted CRISPR‐Cas9 delivery. SNP_SS_ efficiently homes to HCC cells, releases payloads within the glutathione‐rich TME, downregulates *RPS19*, reverses immune suppression, and robustly suppresses HCC progression in vivo. Collectively, the RPS19‐C5aR1‐macrophage axis fuels HCC immune escape, and SNP_SS_‐based *RPS19* gene‐editing offers a promising strategy to remodel the TME and boost HCC immunotherapy efficacy.

## Graphical abstract

By employing in vivo CRISPR functional screening, we identify ribosomal protein RPS19 as a pivotal mediator driving immune evasion in hepatocellular carcinoma, wherein tumor‑released RPS19 binds C5aR1 on tumor‑associated macrophages to initiate downstream immunosuppressive signaling; SP94‑peptide‑modified SNP_SS_ nanoparticles deliver Cas9/sgRPS19 specifically to HCC cells to ablate RPS19, reverse the immunosuppressive tumor microenvironment, reinstate anti‑tumor immune responses and effectively restrain tumor growth.

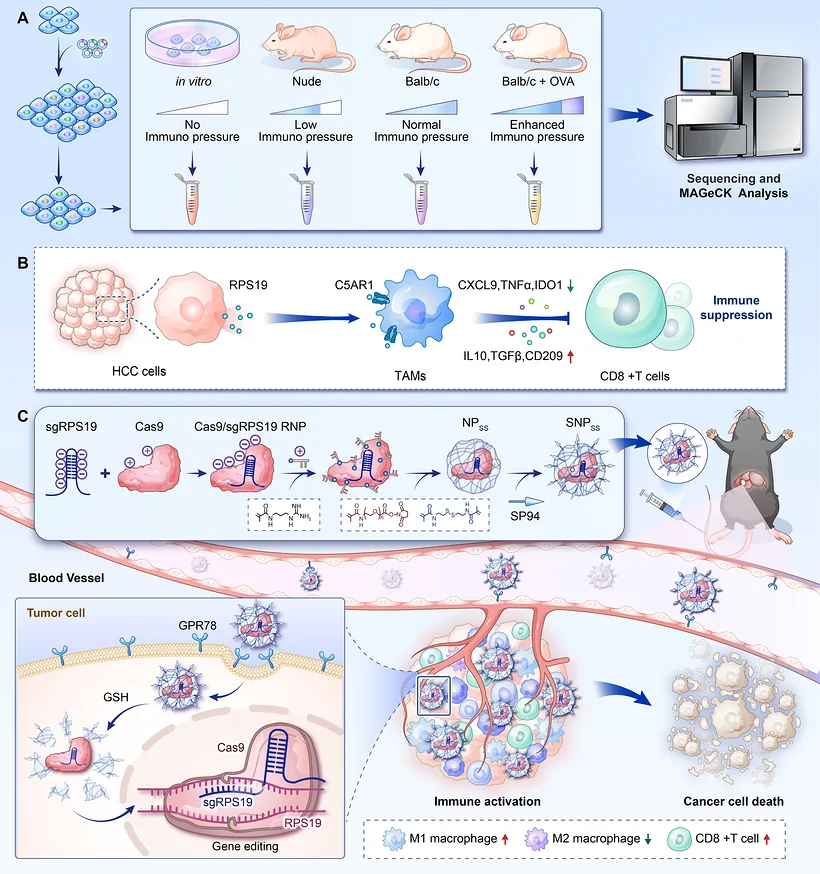

## 1 Introduction

Hepatocellular carcinoma (HCC) is responsible for approximately 80% of all primary liver cancers, making it the sixth leading cause of cancer‐related mortality worldwide [1]. Currently, the first‐line therapies for advanced HCC include sorafenib and lenvatinib; however, these treatments provide only modest benefits, increasing survival by approximately 3 months [2, 3]. Immune checkpoint blockade targeting PD‑1/PD‑L1 has transformed cancer treatment yet yields merely 15% response rates as monotherapy for advanced HCC [4, 5, 6]. Poor treatment outcomes stem from the highly immunosuppressive tumor immune microenvironment [7, 8], where macrophages, Tregs, MDSCs and other dysfunctional immune subsets restrain antitumor immunity and promote HCC progression, relapse and drug resistance [9, 10]. There is an urgent need to investigate the factors driving the accumulation or formation of cells that shape the immunosuppressive TIME in HCC and to identify therapeutic targets for improving prognostic outcomes.

The therapeutic landscape of HCC has undergone a major paradigm shift with the advent of immune‐targeted combination regimens. The phase‐III IMbrave150 trial established atezolizumab plus bevacizumab (T+A) as the benchmark first‐line standard‐of‐care for unresectable HCC, achieving superior survival outcomes compared with sorafenib [11, 12]. Subsequent clinical trials including TALENTACE and TALENTOP further expanded the application of T+A based strategies, exploring combinations with locoregional interventions and peri‐operative therapeutic modalities for high risk HCC patients [13, 14]. Locoregional approaches, including radiofrequency ablation, transarterial chemoembolization (TACE) and radiotherapy, constitute core components of multidisciplinary HCC management for early‐to‐intermediate‐stage disease. A recent meta‐analysis compared radiofrequency ablation and surgical resection for early‐stage HCC: although both strategies yielded comparable 1‐ and 3‐year overall survival rates, ablation is associated with fewer peri‐treatment complications yet a higher local recurrence risk and inferior 3‐year disease‐free survival relative to resection [15]. Nevertheless, intrinsic or therapy‐triggered tumor immune suppression frequently limits durable therapeutic responses following locoregional interventions.

The use of CRISPR/Cas9 for functional genetic screens has emerged as a robust and impartial methodology for identifying novel cancer targets. This technique has also been employed to identify novel tumor immunity modulators, screen for drug targets, discover innovative immuno‐oncology targets, and examine their underlying mechanisms. For example, genome‐wide CRISPR screens conducted in vitro, aided by fluorescence‐activated cell sorting (FACS), have revealed several regulators of PD‐L1 and/or major histocompatibility complex (MHC) class I, which could increase the efficacy of combination immunotherapies for cancer [16, 17]. In subcutaneous tumor models that incorporate various cancer cell lines, combined in vivo screens have pinpointed a critical group of conserved genes, such as Ptpn2, Setdb1, Cop1, Fitm2, and Ripk2, whose absence may enhance ICB immunotherapy [18, 19, 20, 21, 22]. Although orthotopic tumor models may provide a more pertinent environment for investigating targets involved in antitumor immunity, they present significant technical challenges and may not be suitable for genome‐wide screening processes. Consequently, smaller, specialized libraries are often adopted as a more feasible approach for in vivo CRISPR screening. However, to date, no reports of the use of this technology within orthotopic HCC models for the systematic identification and screening of biologically relevant immune targets are available.

In this research, we utilized custom murine CRISPR knockout libraries to perform in vivo CRISPR screens within orthotopic HCC models exhibiting varying levels of host immunity. We identified ribosomal protein S19 (RPS19) as a key regulator of tumor immunity and demonstrated that macrophages represent the critical cell population mediating the immunosuppressive functions of RPS19 in HCC. RPS19 induces an immunosuppressive phenotype in macrophages through binding to C5aR1. Genetic ablation or targeted inhibition of RPS19 in tumor tissues via modified nanoparticles encapsulating the CRISPR‐Cas9 system markedly reversed the immunosuppressive TIME in murine HCC, resulting in tumor regression and prolonged survival. Consequently, our research indicates that RPS19 may represent an overlooked target for immunotherapy, supporting the rationale for a combinatorial approach of RPS19 inhibition in conjunction with ICB immunotherapy.

## 2 Result

### 2.1 An In Vivo CRISPR Screen Identifies RPS19 as a Pivotal Immune Evasion Driver

To systematically identify tumor‐intrinsic genes in HCC cells whose absence may enhances anti‐tumor immunity, we first constructed a murine lentiviral CRISPR‐Cas9 knockout (MusCK) library. This library includes 5 sgRNAs for each of over 4,500 genes implicated in tumor initiation, progression, and immune modulation (Figure S1A and Table S1). We next conducted an in vivo functional CRISPR/Cas9 screen utilizing orthotopic liver tumor models in mice, established with Balb/c background H22 murine hepatocellular carcinoma cells. H22 cells closely resemble human HCC cells and have been used extensively in tumor immunology studies [23, 24]. Our methodology commenced with the artificial expression of membrane‐bound ovalbumin (mOva) in H22 cells, a well‐established technique aimed at augmenting cellular immune responses in syngeneic tumor models [20]. We subsequently transduced H22‐mOva cells with the MusCK library (Figure S1B) and implanted them into the livers of various Balb/c hosts, including Balb/c Foxn1^nu/nu^ (nude) mice that lack T cells, immunocompetent Balb/c mice, and Balb/c hosts that received a vaccination with ovalbumin before transplantation (Figure 1A). Following a fifteen‐day period post‐transplantation, we collected the transplanted cancer cells for subsequent analysis, during which we noted significant variations in tumor growth among the various host types. As expected, immunodeficient Balb/c Foxn1^nu/nu^ hosts presented the largest tumors, whereas the hosts that had been pre‐vaccinated with ovalbumin presented the smallest tumors (Figure 1B–D). After the tumors were harvested, we conducted high‐throughput sequencing of the sgRNA library. The representation of sgRNA in the post‐transplanted cells derived from tumors from both Balb/c+OVA and Balb/c mice was notably different from that in H22 cells cultured in vitro, indicating that the variance was influenced by the immune environment within the host (Figure 1E). Furthermore, principal‐component analysis showed that CRISPR screen samples separated first by in vitro versus in vivo conditions and then by Foxn1^nu/nu^ mice versus immunocompetent mice (Figure 1F). At the same time, the Pearson's correlations of the library distribution from one host type compared to any other type also exhibited high consistency across biological replicates (Figure S1C).

**FIGURE 1:**
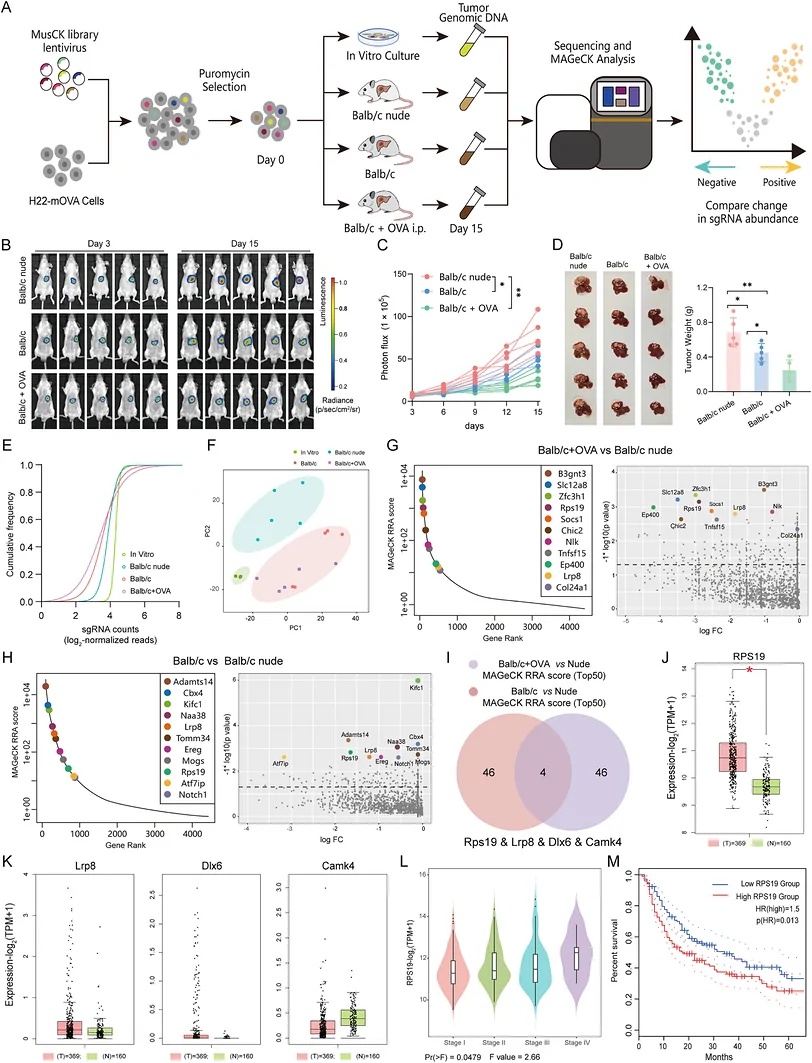
An In Vivo CRISPR Screen Identifies RPS19 as a Pivotal Immune Evasion Driver. (A) Workflow of MusCK in vivo screens to identify the potential targets for immune evasion. i.p., intraperitoneal. (B, C) Luminescence imaging and values of tumors after implantation in the MusCK screens. (D) Mice livers and weight measurements of orthotopic H22‐Luc tumors assessed at 15 days post‐implantation in the MusCK screens. Tumor regions are demarcated by dotted lines (n = 5). (E) Cumulative distribution function graphs showing the library of sgRNA under each condition of the MusCK screens. (F) Principal‐component analysis of sgRNA abundance under each condition of the MusCK screens. (G) Top depleted genes in immunocompetent (Balb/c+OVA boosted) versus immunodeficient (nude) hosts in the MusCK screens. (H) Top depleted genes in immunocompetent (Balb/c wildtype) versus immunodeficient (nude) hosts in the MusCK screens. (I) Venn diagram showing the two selection criteria for identifying candidate genes, specifically those enriched in OVA compared with nude and Balb/c mice versus nude mice. (J, K) The expression levels of 4 candidate genes in liver hepatocellular carcinoma (LIHC, T) and normal liver (N) samples from TCGA database. (L) The expression levels of RPS19 in LIHC tissues from TCGA datasets with different pathological stages. (M) Kaplan‐Meier plot displaying the survival of LIHC patients from TCGA database stratified by high versus low RPS19 expression. *P* values were calculated using two‐tailed unpaired *t* test (D, J, K and L), two‐way ANOVA test (C) and Cox proportional hazards test (M). Data are presented as the means ± SDs (*P < 0.05 and **P < 0.01).

Inspection of the sgRNAs significantly depleted from tumors in Balb/c+OVA hosts (Figure 1G) and wild‐type immunocompetent hosts (Figure 1H) compared with nude immunodeficient mice revealed several mediators involved in immune evasion (*B3gnt3, Socs1 and Cbx4*), confirming the reliability of our screening methods [25, 26, 27, 28]. Next, the examination of the top 50 depleted genes in the MusCK screens, resulting from the comparison of Balb/c+OVA mice with nude mice and Balb/c mice with nude mice, indicated that four genes (*RPS19, Lrp8, Dlx6, and Camk4*) were common to both groups (Figure 1I). To confirm the potential involvement of these candidates in HCC development, we utilized The Cancer Genome Atlas (TCGA) database to investigate the differences in *RPS19*, *Lrp8*, *Dlx6* and *Camk4* expression between liver hepatocellular carcinoma (LIHC) and adjacent normal tissues. The results demonstrated that, in contrast to adjacent tissues, only the expression of RPS19 was significantly upregulated in LIHC tissues (Figure 1J,K). RPS19, which encodes the ribosomal protein S19, is a component of the ribosomal small subunit 40S protein [29]. Analysis of data from TCGA database, stratified according to World Health Organization (WHO) tumor grades I–IV, further indicated that RPS19 expression levels increased progressively with advancing tumor grades (Figure 1L). Additionally, analyses based on TCGA dataset revealed a positive correlation between increased RPS19 expression and unfavorable prognosis in patients with LIHC (Figure 1M). However, since treatment information is not available within the TCGA dataset, we performed further overall survival analysis using the CIDE database, which contains clinical records of patients receiving immunotherapy [30]. Our results revealed that among patients treated with the T+A regimen [12], those with higher RPS19 expression exhibited shorter overall survival compared with patients with low RPS19 expression (Figure S1D). In summary, elevated RPS19 expression is a significant indicator of worse clinical outcomes for HCC patients, regardless of whether they receive immunotherapy. To validate the potential correlation between RPS19 and HCC progression at the protein level, we investigated the association between RPS19 expression and HCC patient prognosis via immunohistochemical (IHC) analysis using 10 adjacent non‐tumor tissues and 40 HCC tissues stratified by WHO histological grades I–IV. The findings once again indicated that the expression of RPS19 was notably higher in HCC samples than in normal tissues, with RPS19 levels increasing with the grade of HCC tumors (Figure S1E,F). The above results suggest the further investigations of RPS19 as a potential candidate therapeutic target for HCC is warranted.

### 2.2 RPS19 Mediated Immunosuppression Plays a Critical Role in HCC Progression

To verify the tumor‐promoting and immunosuppressive roles of RPS19, we performed IHC analysis in 40 HCC specimens. High RPS19 expression correlated with increased Ki67, more CD206^+^ protumor TAMs, and fewer CD8^+^ T cells (Figure S2A,B). Using TCGA LIHC transcriptomic data from 374 patients, GSVA showed that low RPS19 expression enriched immune‐activating pathways including TLR‐NFκB, IL‐6, and IFN‐γ signaling. Conversely, high RPS19 expression was associated with enrichment of immunosuppressive pathways (IL‐10, TGF‐β) and pathways involved in translation and DNA replication. These results link RPS19 to HCC progression and immunosuppression (Figure 2A,C). These findings suggest that RPS19 may facilitate tumor progression by promoting an immunosuppressive microenvironment in HCC.

**FIGURE 2:**
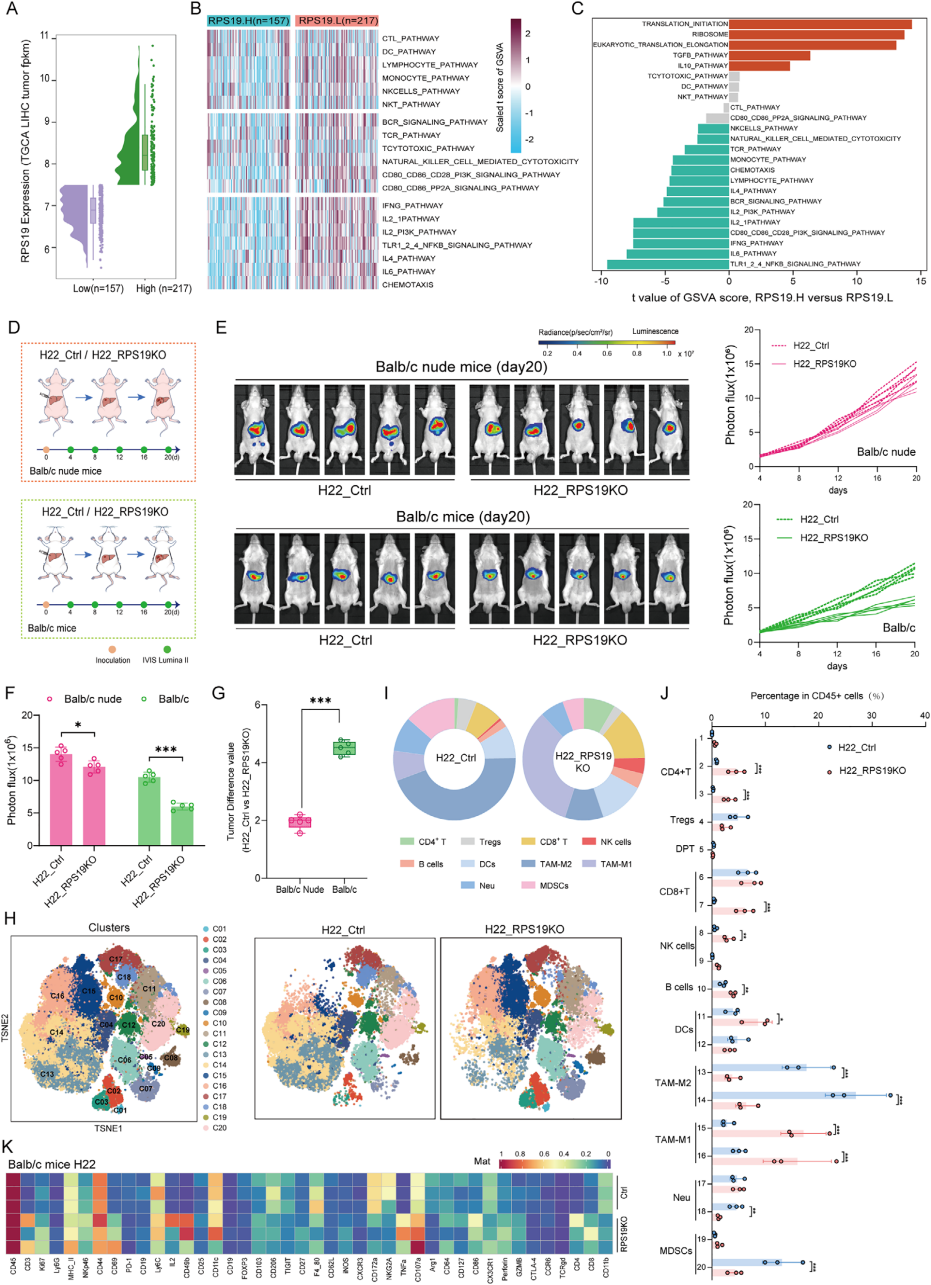
RPS19 Mediated Immunosuppression Plays a Critical Role in HCC Progression. (A) RPS19 expression (fpkm value) in 374 LIHC tissues from TCGA dataset. (B) Heatmap showing the results of pathway enrichments for RPS19‐high and RPS19‐low tissues using gene set variation analysis (GSVA). (C) Differential pathway enriched in RPS19‐high and RPS19‐low tissues by GSVA. Shown are *t* values from a linear model. (D) Diagram depicting the study timeline for observing the in vivo growth of H22_RPS19KO or H22_Ctrl cells in Balb/c nude mice and wild‐type Balb/c hosts (n = 5). (E) The in vivo growth of H22_RPS19KO or H22_Ctrl cells in Balb/c nude mice and wild‐type Balb/c hosts depicted by tumor luminescent signals. (F) The tumor luminescent signals in Balb/c nude mice and wild‐type Balb/c mice bearing orthotopic H22‐Luc liver tumors on Days 20 following cell implantation. (G) The growth disparity between H22_RPS19KO and H22_Ctrl cells in Balb/c nude mice or wild‐type hosts. (H) The *t*‐distributed stochastic neighbor embedding (*t*‐SNE) visualization revealed 20 distinct immune cell clusters identified within the CD45^+^ tumor‐infiltrating leukocytes derived from H22_RPS19KO tumors and H22_Ctrl tumors of wildtype Balb/c miceusing single‐cell mass cytometry (CyTOF) analysis (n = 3, left: pooled data, right: H22_RPS19KO tumor *vs* H22_Ctrl tumor). (I) The proportion of each cell type in H22_RPS19KO tumors and H22_Ctrl tumors. (J) The proportional discrepancy among the 20 identified cell clusters by CyTOF in H22_RPS19KO tumors versus H22_Ctrl tumors. (K) The heatmap illustrates the expression levels of 42 markers in the tumor‐infiltrating immune cells from Balb/c mice bearing orthotopic H22 tumors (designated as RPS19 Ctrl for the upper 3 rows and RPS19 KO for the lower 3 rows). *P* values were calculated using two‐sided unpaired limma‐moderated *t* test (C) and two‐tailed unpaired *t* tests (F, G and J). Data are presented as the means ± SDs (*P < 0.05, **P < 0.01, and ***P < 0.001).

To more rigorously illustrate that RPS19 affects HCC progression and its immunosuppressive properties, we disrupted the RPS19 gene via CRISPR‐Cas9 lentiviral transduction in Hepa1‐6 and H22 mouse hepatoma cell lines, which are derived from distinct genetic backgrounds [31]. RPS19‐knockout cells were subjected to monoclonalization, and cell clones with complete RPS19 knockout were validated using western blotting and sanger sequencing (Figure S2C,D). Subsequently, the in vitro growth of RPS19‐knockout cells was monitored using the Agilent xCELLigence real‐time cell analysis (RTCA) system and Cell Counting Kit‐8 analysis. We observed that RPS19 disruption impeded the growth of both Hepa1‐6 and H22 cells by approximately 20% after 96 h of culture (Figure S2E,F). Next, RPS19‐knockout H22 cells (H22_RPS19KO) or control H22 cells (H22_Ctrl) were orthotopically inoculated into the livers of syngeneic Balb/c Foxn1^nu/nu^ nude and wild‐type Balb/c hosts. Orthotopic tumor growth was assessed by measuring luminescence intensity using an IVIS Lumina II imaging system (Figure 2D). Notably, in both Balb/c nude mice and immunocompetent wild‐type Balb/c mice, H22_RPS19KO cells exhibited attenuated tumor growth compared with H22_Ctrl cells, as demonstrated by the pronounced reduction in bioluminescence signal intensity (Figure 2E,F). However, the difference in tumor growth between H22_RPS19KO and H22_Ctrl cells was significantly greater in immunocompetent wildtype hosts than in nude hosts (Figure 2G). We then replicated the aforementioned experiments by orthotopically implanting either RPS19‐knockout Hepa1‐6 cells (Hepa1‐6_RPS19KO) or control Hepa1‐6 cells (Hepa1‐6_Ctrl) into the livers of syngeneic Rag1^−/−^ immunodeficient mice (a C57BL/6 background strain) or immunocompetent wild‐type C57BL/6 mice. Consistently, the growth disparity between Hepa1‐6_RPS19KO and Hepa1‐6_Ctrl cells was more pronounced in immunocompetent hosts than in Rag1^−/−^ immunodeficient hosts (Figure S2G–L). The foregoing results implies that, besides promoting hepatocellular carcinoma cell proliferation, RPS19‐mediated modulation of the immune microenvironment also plays a critical role in HCC progression.

To further investigated the potential impact of RPS19 on the HCC immune microenvironment, we additionally conducted a single‐cell mass cytometry (CyTOF) analysis of tumor tissues derived from H22_RPS19KO and H22_Ctrl cells inoculated wildtype Balb/c mice. At the single‐cell level, CyTOF utilizes 42 monoclonal antibodies (mAbs) to identify immune cell lineages and assess the functional molecules present in tumor‐infiltrating leukocytes (TILs). An assessment of the CD45^+^ cell population revealed their segregation into a total of 20 distinct clusters (Figure 2H). Further analysis of the differences across these 20 clusters between the H22_RPS19KO and H22_Ctrl tumors revealed a significant increase in the expansion of CD4^+^ T cells (Clusters 2 and 3), CD8^+^ effector/memory T cells (Cluster 7), NK cells (Cluster 8), B cells (Cluster 10), DCs (Cluster 11), and M1 macrophages (Clusters 15 and 16) within the H22_RPS19KO tumor. Conversely, substantial augmentation in the numbers of M2 macrophages (Clusters 13 and 14), neutrophils (Cluster 18), and MDSCs (Cluster 20) were noted in the H22_Ctrl tumor (Figure 2I,J). Meanwhile, CyTOF analysis unveiled alterations in key immune cell markers within TILs between H22_RPS19KO and H22_Ctrl tumor tissues. For example, the expression levels of an activated T cells marker CD69 [32, 33], the immunostimulatory cytokines IL‐2 and TNF‐α [34], the cytotoxic cytokines GZMB and perforin, activated NK cell markers CD49b and NKp46 [35], and M1 macrophages markers CD86 and iNOS [36] were significantly upregulated in H22_RPS19KO tumor tissues compared with those in H22_Ctrl tumor tissues. In contrast, marker molecules and functional molecules of suppressive immune cells, including M2 macrophage markers (CD206, CD172a, Arg1) and myeloid‐derived suppressor cells (MDSCs) marker CD11b, were significantly upregulated in H22_Ctrl tumor tissues (Figure 2K and Figure S3). Subsequently, flow cytometry analysis confirmed that the TIME of H22‐RPS19KO tumor tissues exhibited a significant increase in the proportions of activated T cells, activated NK cells, and anti‐tumor M1 macrophages, along with a significant decrease in the proportions of pro‐tumor M2 macrophages and MDSCs, compared with the TIME of H22‐Ctrl tumor tissues. Among these changes, the alteration in macrophage subsets was the most prominent (Figure S4). These analyses further suggest that RPS19‐mediated suppression of antitumor immune responses in vivo constitutes a critical mechanism underlying its promotion of HCC progression.

### 2.3 scRNA‐seq Analysis Predicts Macrophages Represent a Critical Cell Subset Mediating the Suppressive Effects of RPS19 in HCC

It is well established that the TIME is a major contributing factor of tumor progression, relapse, metastasis and even drug resistance [37]. However, the tumor immune microenvironment (TIME) is a complex network of immune and stromal cells. To further confirm how RPS19 regulates the TIME and identify key cell subsets involved, we obtained a single‐cell RNA‐sequencing dataset from the Genome Sequence Archive (BioProject ID: PRJCA00774432) [38]. This study performed scRNA‐seq on 124 patient tumor samples, 79 of which had HCC. HCC TIME was stratified into immune‐activated and immune‐suppressive subtypes. Activated samples were divided into IA and IR; suppressive ones into ISS, ISM, and IE (Figure 3A). As our in vivo screening used nude mice lacking mature T cells, we selected IA and IE subtypes for analysis: IA matches the relevant immune profile, while IE represents typical immunosuppressive ‘cold’ tumors. Unsupervised clustering was performed on 157 840 cells from 8 IA and 8 IE samples. A total of 31 cell clusters were identified and were annotated into 9 cell subsets according to markers genes (Figure 3A and Figure S5A). Copy number variations (CNV) analysis revealed that most annotated hepatocytes were malignant cells (Figure S5B). Subsequently, we compared the expression of RPS19 in the total cell populations of these two subtypes, as well as its expression in hepatocytes derived from these subtypes. In both the total cell population and hepatocyte subsets, RPS19 expression was significantly higher in IE subtype than in IA subtype (Figure 3B). In the cell composition analysis, IE tissues, as expected, exhibited a notable reduction in T and B cells and a marked increase in macrophages compared with IA tissues. No significant differences in other myeloid cell populations were observed between the two tissue subtypes (Figure 3C,D). Moreover, macrophages in the IE subtype exhibited a more pronounced protumor phenotype, characterized by high expression of protumor genes (e.g., *TREM2*, *VEGFA*, *MRC1* and *TGFB1*) and low expression of tumor‐suppressive molecules (e.g., *TNF*, *INOS*, *CXCL9* and *CXCL10*). Consistently, T and NK cells in the IA subtype displayed a more activated phenotype compared with those in the IE subtype (Figure 3E). The results of differential genes (DEGs) analysis between the two subtypes further confirm the immune activated phenotype of the IA tissues and the immunosuppressive phenotype of the IE tissues (Figure 3F). Further, we aim to identify the key cell population mediating the immunosuppressive function of RPS19 in HCC using the receptor of RPS19.

**FIGURE 3:**
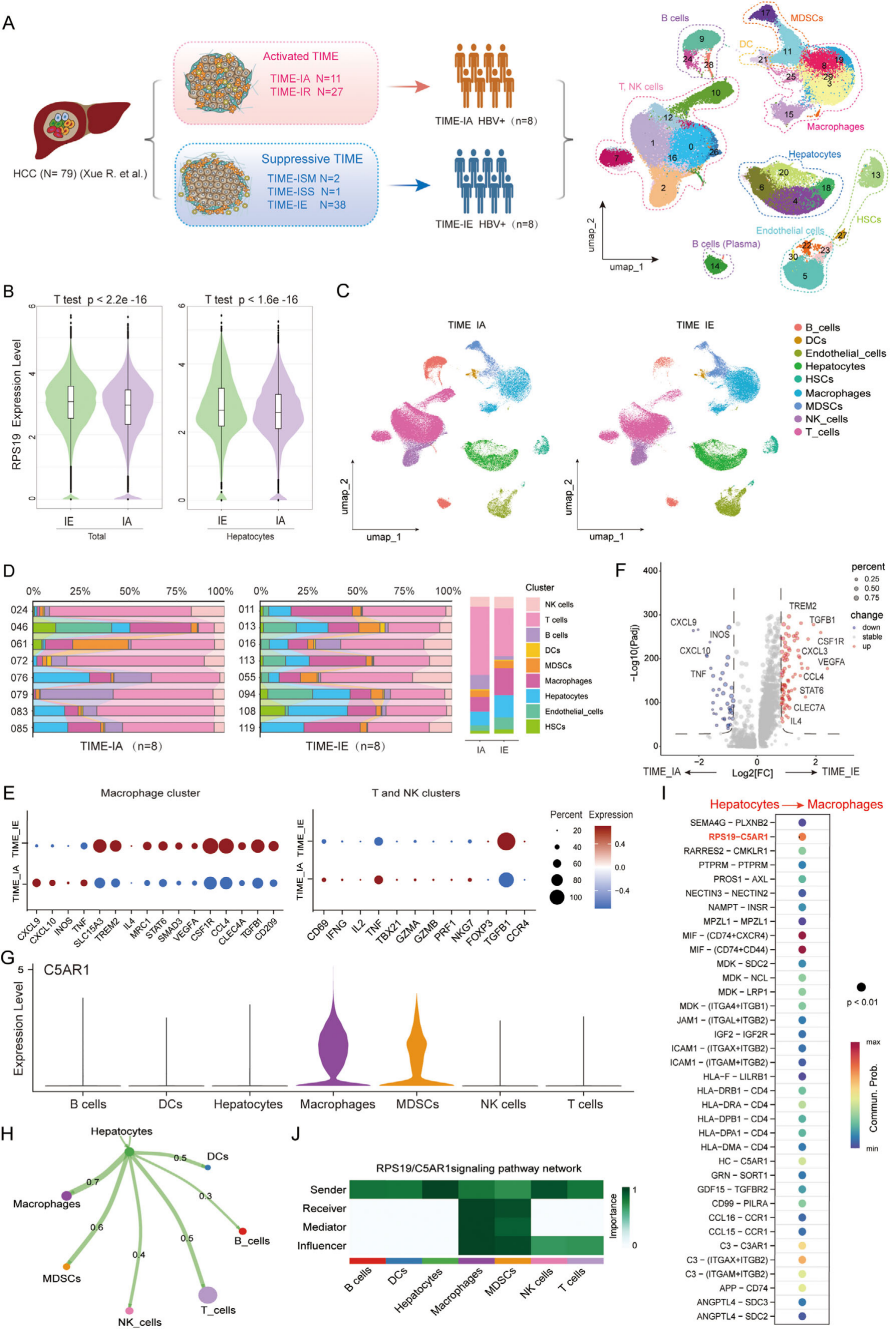
scRNA‐seq Analysis Predicts Macrophages Represent a Critical Cell Subset Mediating the Suppressive Effects of RPS19 in HCC. (A) The numbers of HCC cases from Genome Sequence Archive at the National Genomics Data Center (Beijing,China) under the BioProject ID PRJCA007744 and samples collected for each cancer type are denoted (left). Uniform manifold approximation and projection (UMAP) plot showing the major cell types of randomly selected 8 IA and 8 IE samples. Dots represent individual cells, and colors represent different cell populations (right). (B) Violin plot showing the expression level of RPS19 in the total cells and hepatocytes of IA and IE groups. (C) UMAP plots showing the annotated cell types in immune activation (IA) and immune exclusion (IE) groups. (D) Stacked bar graph showing the proportions of the annotated cell types in groups IA and IE. (E) The expression of specific cytokines, chemokines and receptors in macrophages and T cells (including NK cells) across IA and IE groups. (F) Volcano plot showing differentially expressed genes between IA and IE total cells (Smart‐seq2 scRNA‐seq). (G) Violin plot showing the expression level of C5AR1 across the annotated cell types of all samples. (H) Circle diagram of CellChat analysis showing the strength of the cell‐cell communication from the hepatocytes to immune cells. (I) Multiple ligand‐receptor pairs analysis of CellChat showing the communication probabilities mediated by ligand‐receptor pairs from hepatocytes to macrophages. (J) Heatmap presenting the importance score of hepatocytes and immune cells (sender, receiver, mediator, and influencer) in the RPS19‐C5AR1 signaling pathway. *P* values were calculated using two‐sided unpaired limma moderated *t* test; two‐tailed unpaired *t* tests (B), log2 (fold change) R 1.5 (F) and one‐sided permutation test (I).

According to previous publications [39, 40], RPS19 is the only other known endogenous ligand of eukaryotic origin, aside from complement C5a, that binds to C5aR1. However, there remains significant controversy regarding the specific cell types that RPS19, released from tumor cells, ultimately targets via C5aR1, as well as the functions it exerts [40, 41, 42]. We analyzed the expression of C5aR1 across annotated cell populations in these 16 HCC tissues and observed that only macrophages and MDSCs express C5aR1 (Figure 3G). We next added RPS19‐C5aR1 ligand‐receptor pair into CellChatDB of CellChat R package and employed CellChat to systematically interrogate cellular communications based on cognate ligand and receptor co‐expression between cell types. First, we found that globally, macrophages had the most outgoing and incoming signaling contributions in the TIME (Figure S5C). Next, our analysis revealed that the most robust interaction strength occurs between hepatocytes and macrophages (Figure 3H). More important, among the identified ligand‐receptor pairs mediated communication from hepatocytes to macrophages, the communication probabilities of RPS19‐C5aR1 ranks second, surpassed only by the MIF pathway (Figure 3I). Multiplex immunofluorescence staining was performed to validate the spatial prerequisite for RPS19‐C5AR1 crosstalk predicted by CellChat analysis. In orthotopic mouse HCC tissues, RPS19 and C5AR1 exhibited substantial spatial overlap, which was further validated by fluorescence line‐scan profiles (Figure S6A). Consistent with these findings, obvious spatial proximity of RPS19 and C5AR1 was also observed in human HCC specimens, despite slightly lower overall signal intensity (Figure S6B). These in situ findings reveal spatial proximity between RPS19 and C5aR1 in mouse and human HCC, supporting RPS19‐C5aR1 crosstalk in the tumour microenvironment. Network centrality analysis further predicted that the hepatocytes subset was the most important sender in the RPS19‐C5aR1 signaling, while macrophages emerged as the most prominent receiver (Figure 3J). To investigate whether RPS19 acts on discrete TAM sub‐clusters rather than bulk macrophages, we analysed scRNA‐seq data from IA and IE HCC groups. Dot‐plot analysis confirmed broad upregulation of RPS19 across multiple cell types in the IE group (Figure S7A). Unbiased re‐clustering resolved five TAM sub‐clusters (Mac_0‐Mac_4), with Mac_0 showing the most pronounced compositional shifts between IA and IE (Figure S7B–D). C5aR1 expression was markedly higher in Mac_0 of IE tumours (Figure S7E). Within Mac_0, high RPS19 suppressed pro‐inflammatory TNF and CXCL9, and induced immunosuppressive TGFβ1 and CD209, whereas other macrophage subsets showed limited transcriptional perturbation (Figure S7F). CellChat predicted strengthened hepatocyte‐macrophage signalling in IE lesions, supporting Mac_0‐centred immune suppression downstream of RPS19‐C5aR1 signalling (Figure S7G,H). These findings indicate that Mac_0 TAMs constitute the primary target of RPS19, which exerts immunosuppressive functions through the RPS19‐C5aR1 signalling axis.

Given that C5aR1 is expressed on macrophage surfaces, RPS19 must be released from tumor cells to engage C5aR1. To clarify extracellular RPS19 release, we cultured Hepa1‐6 tumor cells with primary CD8^+^ T cells. Monocultures produced negligible supernatant RPS19, while robust RPS19 accumulated upon co‐culture of RPS19‐WT tumor cells with CD8^+^ T cells. This signal was greatly diminished using RPS19‐KO tumor cells, with residual RPS19 presumably derived from damaged CD8^+^ T cells (Figure S8A). Comparable LDH release in both co‐culture groups showed RPS19 deletion does not affect tumor susceptibility to CD8^+^ T‐cell‐mediated lysis (Figure S8B). After ultracentrifugation, abundant RPS19 remained in exosome‐depleted supernatant, yet no RPS19 was detectable in exosome pellets (Figure S8C,D). Collectively, RPS19 passively leaks from CD8^+^ T‐cell‐injured tumor cells rather than being exosomally secreted. Based on the comprehensive analyses above, we hypothesize that macrophages represent the most critical cell subset mediating the suppressive effects of RPS19 within the TIME of HCC.

### 2.4 RPS19 Promotes HCC Progression by Inducing Immunosuppression in Macrophages via C5aR1

Macrophages, a well‐recognized subset of innate immune cells, exhibit remarkable epigenomic and transcriptomic plasticity driven, in part, by changes in the microenvironment [43, 44]. Tumor associated macrophages (TAMs) can contribute to tumor clearance or protection based on their polarization into macrophages that mediate anti‐tumor/pro‐inflammatory or pro‐tumor effects, respectively [45, 46]. Our prior bioinformatic analyses suggested that RPS19 mediates immunomodulatory effects through binding to macrophage‐surface C5aR1. However, C5aR1 possesses the canonical ligand C5a, which is widespread present in vivo [47]. We compared the binding properties of RPS19 and C5a toward C5aR1 using ELISA‐based binding assays. Both ligands bound C5aR1 in a concentration‐dependent manner with similar binding capacity (Figure S9A,B). Reciprocal competitive‐binding assays showed that RPS19 and C5a mutually displace one another, confirming competition for C5aR1 (Figure S9C,D). We next quantified RPS19 and C5a in tumour interstitial fluid (TIF) from orthotopic Hepa1‐6 tumours [48]. While TIF RPS19 was greatly decreased in RPS19‐KO tumours, TIF C5a levels remained unchanged between RPS19‐WT and RPS19‐KO groups (Figure S9E). These data suggest that RPS19 competes with C5a for C5aR1, and that RPS19 ablation does not induce compensatory C5a upregulation in the HCC tumour microenvironment, further establishing RPS19 as a C5a‐independent immunomodulatory molecule.

To rigorously validate that RPS19 induces tumor immunosuppression via macrophages, we first assessed the impact of RPS19 on macrophage polarization. We treated human THP‐1 macrophages with human RPS19 under either M1‐polarization (stimulated with LPS and IFN‐γ) or M2‐polarization (stimulated with IL‐4 and IL‐13) conditions. Flow cytometry and qRT‐PCR analyses demonstrated that RPS19 abrogated the antitumor phenotypes of THP‐1 macrophages under M1‐polarization conditions, as evidenced by reduced expression of TNFα, INOS, and CXCL9 (Figure 4A–C and Figure S10A). Conversely, under M2‐polarization conditions, RPS19 enhanced the protumor phenotypes of these cells, characterized by upregulated expression of IL10, CD209, and TGFβ (Figure 4D–F and Figure S10B). Notably, these molecules were among the signature genes of the macrophage cluster identified in RPS19‐high‐expressing IE tissues in our preceding scRNA‐seq analysis (Figure 3D,E). Furthermore, RNA‐seq analysis of THP‐1 macrophages treated with RPS19 versus controls revealed that a panel of protumor molecules, including VEGFA, TGFB, and MMP14, were upregulated, whereas several antitumor molecules, including CXCL9, CXCL10, and NOS2, were downregulated (Figure 4G). Pathway enrichment analysis of the DEGs uncovered the enrichment of several protumor pathways associated with angiogenesis and epithelial‐to‐mesenchymal transition (EMT), as well as pathways linked to protumor macrophage polarization, such as the ERK cascade (Figure 4H). Beyond THP‐1 macrophages, similar effects were observed with human RPS19 in human peripheral blood macrophages (Figure S10C–E) and with mouse RPS19 in mouse RAW264.7 macrophages (Figure S10F). Subsequently, we evaluated the impact of RPS19 on the activation of CD8^+^ T cells isolated from human peripheral blood (Figure S10G). We observed that RPS19 alone exerts no effect on CD8^+^ T cell activation; however, in co‐cultures of peripheral blood macrophages and CD8^+^ T cells, RPS19 significantly inhibited CD8^+^ T cell activation, as evidenced by the downregulated expression of GZMB, perforin, and IFN‐γ (Figure 4I,J). More importantly, in wild‐type C57BL/6 mice depleted of macrophages using clodronate liposomes [49], the growth discrepancy between Hepa1‐6_RPS19KO and Hepa1‐6_Ctrl cells were markedly attenuated compared with that observed in untreated wild‐type C57BL/6 mice (Figure 4K,L).

**FIGURE 4:**
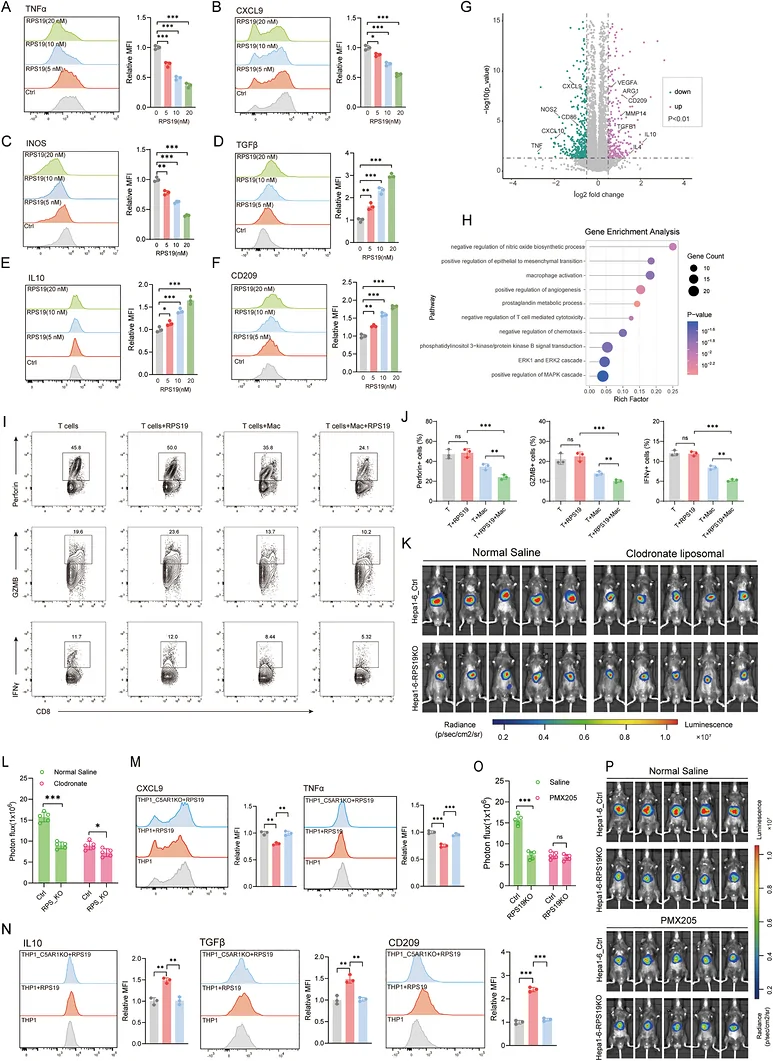
RPS19 promotes HCC progression by inducing immunosuppression in macrophages via C5AR1. (A‐C) Flow cytometry analysis of TNF, CXCL9 and INOS expression in RPS19‐treated THP‐1 macrophages under M1‐polarization conditions. (D‐F) Flow cytometry analysis of IL‐10, CD209, and TGF‐β expression in RPS19‐treated THP‐1 macrophages under M2‐polarization conditions. (G) Volcano plot illustrating differentially expressed genes (DEGs) in THP‐1 macrophages following treatment with RPS19 or control protein. (H) Pathway enrichment analysis of DEGs between RPS19‐treated and control‐treated THP‐1 macrophages. (I, J) Flow cytometry analysis of activation in TCR‐stimulated human peripheral blood CD8^+^ T cells under the indicated treatment conditions (Mac: human peripheral blood macrophages). (K, L) Tumor growth of Hepa1‐6_RPS19KO and Hepa1‐6_Ctrl cells in wild‐type C57BL/6 mice or C57BL/6 mice with pre‐depleted macrophages via clodronate liposomes. (M) Expression levels of CXCL9 and TNF‐α in control knockout THP‐1 macrophages (THP‐1) or C5AR1‐knockout THP‐1 macrophages (THP1_C5AR1KO) following RPS19 treatment under M1‐polarization conditions. (N) Expression levels of IL‐10, TGF‐β, and CD209 in control knockout THP‐1 macrophages or C5AR1‐knockout THP‐1 macrophages (THP1_C5AR1KO) following RPS19 treatment under M2‐polarization conditions (O, P) Tumor growth of Hepa1‐6_RPS19KO and Hepa1‐6_Ctrl cells in wild‐type C57BL/6 mice or C57BL/6 mice treated with PMX205 (a C5AR1 inhibitor). *P* values were calculated using two‐sided unpaired t‐tests (A–G, J, L–O) and Fisher's exact test with Benjamini–Hochberg multiple‐test correction (H). Data are presented as the means ± SDs (ns: no significant, *P < 0.05, **P < 0.01, and ***P < 0.001).

Our preceding scRNA‐seq analysis predicted that RPS19‐C5aR1 constitutes a key ligand‐receptor pair mediating crosstalk from malignant hepatocytes to macrophages. Accordingly, we confirmed high C5aR1 expression in human peripheral blood‐derived macrophages, but not in human CD4^+^ T cells, CD8^+^ T cells, natural killer (NK) cells, or B cells (Figure S11A,B). C5aR1 is also highly expressed in THP‐1 and RAW264.7 cells, we then knocked out C5aR1 expression in THP‐1 cells (Figure S11C,D) and subsequently evaluated the effects of RPS19 on these C5aR1‐deficient macrophages. As anticipated, C5aR1 deficiency significantly abrogated the effects of RPS19 on THP‐1 cell polarization under both M1 and M2 polarizing conditions (Figure 4M,N). Concordantly, the C5aR1 inhibitor PMX205 exerted a similar abrogating effect on the actions of human RPS19 in THP‐1 macrophages (Figure S12A), as well as on peripheral blood‐derived macrophages (Figure S12B). In wild‐type C57BL/6 mice, we observed that the growth difference between Hepa1‐6_RPS19KO and Hepa1‐6_Ctrl cells was also attenuated by PMX205 (Figure 4O,P). Collectively, these findings confirm that macrophages with high C5aR1 expression play a critical role in mediating the immunosuppressive effects of RPS19 within the TIME of hepatocellular carcinoma HCC.

### 2.5 SP94 Peptide‐Modified Nanoparticles Facilitate Delivery of the CRISPR‐Cas9 System to the HCC Tumor Site

Our study identifies RPS19 as a promising target for HCC immunotherapy, as it reshapes the tumor immune microenvironment and drives HCC progression. Genome‐editing‐mediated RPS19 inhibition in tumor cells offers higher specificity and longer efficacy than conventional immunotherapies such as adoptive cell transfer and immune checkpoint blockade. However, CRISPR‐Cas9 delivery via viral vectors suffers from poor specificity and safety concerns. To overcome these limitations, we developed disulfide bond‐crosslinked nanoparticles loaded with Cas9 and RPS19‐targeting sgRNA, which enable targeted release in the high‐GSH tumor microenvironment [50]. Furthermore, we cross‐linked the nanoparticles with polyethylene glycol (PEG) decorated with the HCC‐specific targeting peptide SP94 to further enhance their tumor‐targeting capability, and this modified formulation was designated as SNP_SS_(Cas9/sgRPS19) [51] (Figure 5A). A dynamic light scattering (DLS) analysis was conducted to assess the nanoparticle size, revealing that SNP_SS_(Cas9/sgRPS19) had a size of 109 nm and a polydispersity index (PDI) of 0.13 (Figure 5B). Zeta potential measurements indicated that SNP_SS_(Cas9/sgRPS19) had a value of approximately +27.43 ± 1.65 mV (Figure 5C). Then, we employed transmission electron microscopy (TEM) to detect the morphology of the SNP_SS_(Cas9/sgRPS19), confirming their spherical structure. Notably, the nanoparticles exhibited rapid degradation and the subsequent release of Cas9/sgRPS19 in a reducing intracellular environment mimicking elevated levels of GSH in tumor microenvironment. This effect was absent in the nonreducing environment (Figure 5D). We assessed the editing efficacy of the RPS19 gene and evaluated the protective effect of our CRISPR/Cas9 nanoparticles on the sgRNA by performing T7 endonuclease I (T7E1) cleavage assays [52]. Remarkably, the presence of RNase did not affect the gene editing efficiency of SNP_SS_(Cas9/sgRPS19), whereas the addition of RNase negatively affected the DNA cleavage ability of free Cas9/sgRPS19 (Figure 5E). We subsequently explored the effectiveness of utilizing nanoparticles for the delivery of Cas9/sgRPS19 by performing in vitro gene editing assays. The nanoparticles were introduced into mouse HCC cells (Hepa1‐6), and we assessed the disruption efficiency and specificity of the RPS19 gene by SNP_SS_(Cas9/sgRPS19) using Sanger sequencing. Our findings revealed that the CRISPR/Cas9 editing site was located 3–5 bases upstream of the protospacer adjacent motif (PAM) sequence (Figure 5F). Concurrent flow cytometric analysis further demonstrated that SNP_SS_(Cas9/sgRPS19) nanoparticles mediated RPS19 disruption in more than 90% of the cells (Figure 5G,H). Next, Cas9 was labeled with Cy5.5 and sgRPS19 with FITC, then encapsulated into nanoparticles for HCC targeting evaluation. Confocal imaging showed that SNP_SS_(Cas9/sgRPS19) exhibited much higher targeting efficiency in Hepa1‐6 cells than non‐targeted NP_SS_(Cas9/sgRPS19) (Figure 5I). We investigated the in vivo biodistribution of nanoparticles in orthotopic Hepa1‐6 tumor‐bearing mice. Fluorescence imaging showed that SNP_SS_(Cas9/sgRPS19) produced significantly stronger fluorescence signals in liver tumors than non‐targeted NP_SS_(Cas9/sgRPS19) (Figure 5J). Furthermore, the degradation rate of Cy5.5 in the liver was markedly slower in the SNP_SS_(Cas9/sgRPS19) group than in the NP_SS_(Cas9/sgRPS19) group over a 24‐hour period. In ex vivo imaging of mouse livers, the Cy5.5 fluorescent signal at tumor sites was also more prominent in the SNP_SS_(Cas9/sgRPS19) group than in the NP_SS_(Cas9/sgRPS19) group (Figure 5K,L). Confocal imaging further confirmed that SNP_SS_(Cas9/sgRPS19) targeted HCC cells more effectively and accumulated within the tumor, indicating its potential for precise tumor targeting (Figure 5M).

**FIGURE 5:**
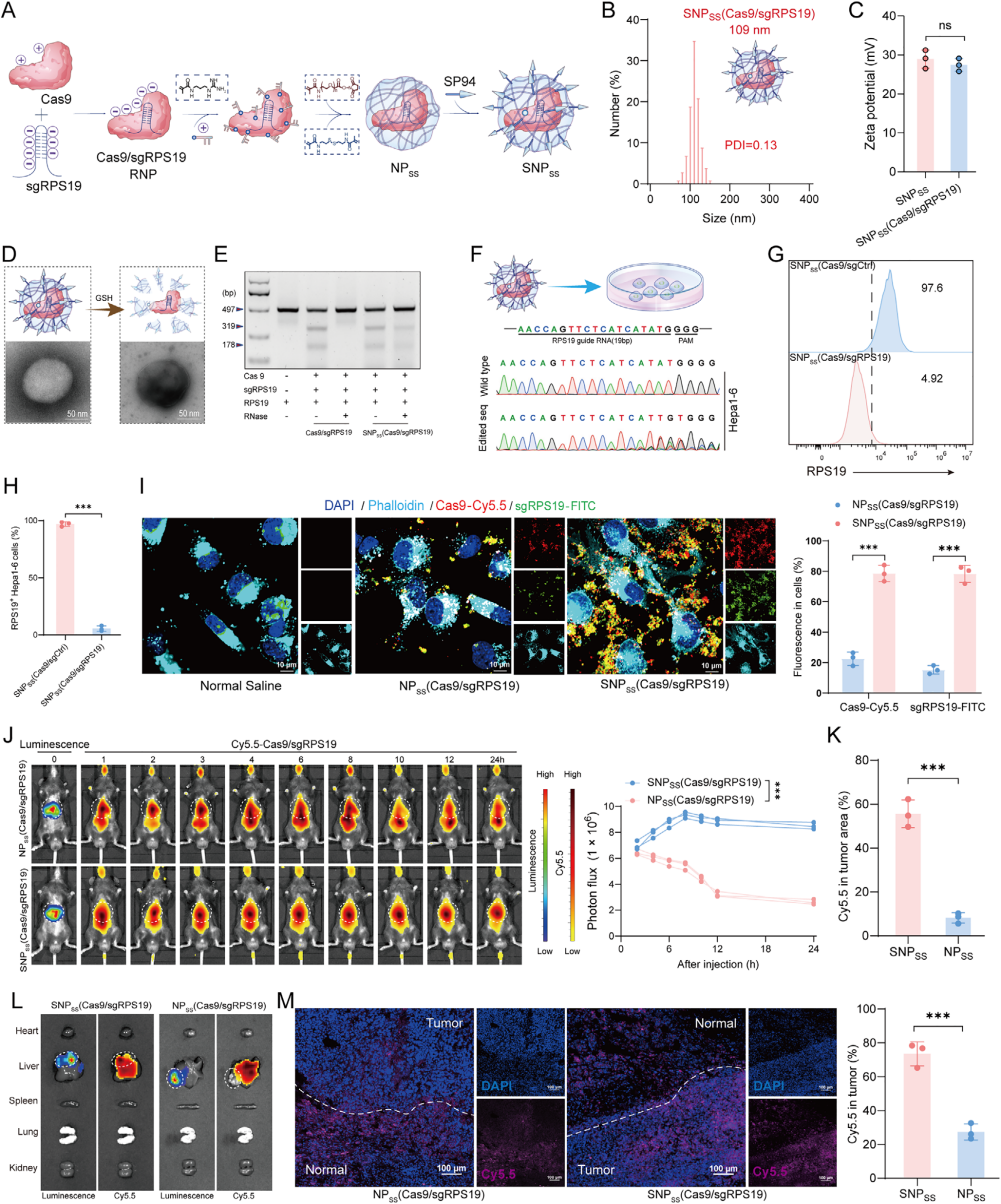
SP94 Peptide‐Modified Nanoparticles Facilitate Delivery of the CRISPR‐Cas9 System to the HCC Tumor Site. (A) Schematic diagram illustrating the construction of nanoparticles for targeted delivery of the CRISPR‐Cas9 system to HCC. (B) DLS image showing the particle size of the SNP_SS_(Cas9/sgRPS19). (C) Zeta potential of SNP_SS_(Cas9/sgRPS19). (D) Transmission electron microscopy images displaying the spherical morphology of the SNP_SS_(Cas9/sgRPS19) in saline with and without glutathione (GSH). (E) Agarose gel electrophoresis for detecting indel formation in RPS19 DNA following treatment with SNP_SS_ (Cas9/sgRPS19) or other specified treatments, with or without RNase intervention. (F) DNA sequencing validating RPS19 gene editing in Hepa1‐6 cells following treatment with SNP_SS_ (Cas9/sgRPS19). (G and H) Flow cytometry analysis validating RPS19 protein degradation in Hepa1‐6 cells following treatment with SNP_SS_ (Cas9/sgRPS19). (I) Images of immunofluorescence staining depicting the internalization of NP_SS_(Cas9/sgRNA) and SNP_SS_(Cas9/sgRNA) into Hepa1‐6 cells. Scale bar: 10 µm. (J) Fluorescence images of C57BL/6 mice bearing orthotopic Hepa1‐6‐Luc tumors following intravenous administration of NP_SS_ (Cas9/sgRPS19) or SNP_SS_ (Cas9/sgRPS19). (K and L) Measurements of luciferase luminescence and Cy5.5‐Cas9 fluorescence in the heart, liver, spleen, lung, and kidney of C57BL/6 mice bearing orthotopic Hepa1‐6‐Luc tumors at 24 h following intravenous injection of NP_SS_ (Cas9/sgRPS19) or SNP_SS_ (Cas9/sgRPS19). (M) Confocal microscopy images illustrating the penetration of NP_SS_(Cas9/sgRPS19) and SNP_SS_(Cas9/sgRPS19) into the tumor regions. Scale bars: 100 µm. *P* values were calculated using two‐tailed unpaired *t* tests (C, H, I, K and M) or two‐way ANOVA (J). Data are presented as the means ± SDs (ns: not significant, *P < 0.05, **P < 0.01, and ***P < 0.001).

Spontaneous HCC, induced by transposon systems containing plasmids that encode oncogenes and transposases, may provide a more appropriate context for the study of multiple tumor microenvironments [53]. Consequently, we further developed a spontaneous HCC model through a hydrodynamic tail vein injection of plasmids encoding myr‐AKT1‐luc and N‐RasV12, accompanied by Sleeping Beauty transposase, to facilitate spontaneous HCC development in mice [54]. The myr‐AKT1‐luc plasmid also incorporates a segment of the luciferase gene, allowing us to monitor the liver tumor size in the mice using the IVIS Spectrum system. Through these methodologies, we successfully established a user‐friendly bioluminescence‐based spontaneous HCC model to evaluate the therapeutic effects of CRISPR/Cas9 nanoparticles. Similarly, SNP_SS_(Cas9/sgRPS19) presented superior tumor targeting in the spontaneous HCC model compared with NP_SS_(Cas9/sgRPS19) (Figure S13A–C). These findings suggest that the SP94 polypeptide efficiently facilitates the clustering of nanoparticles within HCC tissues in vivo, highlighting its potential for in situ gene editing within tumors.

### 2.6 Targeting Tumor RPS19 via SNP_SS_(Cas9/sgRPS19) Nanoparticles Remodels the TIME and Effectively Suppresses HCC Progression

We evaluated the therapeutic efficacy of SNP_SS_(Cas9/sgRPS19) by establishing an orthotopic HCC mouse model with Hepa1‐6‐luc cells. The mice were randomly assigned to various treatment groups and received intravenous injections of saline, SNP_SS_(Cas9/sgCtrl), or SNP_SS_(Cas9/sgRPS19) every 5 days (Figure 6A). Notably, the group treated with SNP_SS_(Cas9/sgRPS19) presented a significant reduction in tumor growth, as evidenced by a marked decrease in the intensity of the bioluminescent signal. Conversely, the mice that were administered normal saline or SNP_SS_(Cas9/sgCtrl) presented an increase in bioluminescent signal intensity, indicating that these interventions did not effectively impede tumor growth (Figure 6B). Following 4 administrations of the treatment, the mice were euthanized for dissection. The findings indicated that both tumor volume and weight were considerably lower in the SNP_SS_(Cas9/sgRPS19) group than in the saline or SNP_SS_(Cas9/sgCtrl) groups (Figure 6C). An analysis of the survival indicated a notable improvement in the median survival time, exceeding 70 days for the SNP_SS_(Cas9/sgRPS19) treatment group and 47 and 43 days for the saline and SNP_SS_(Cas9/sgCtrl) groups, respectively (Figure 6D). Tumor samples from mice administered SNP_SS_(Cas9/sgRPS19) or the two control treatments were analyzed on Day 25 using next‐generation sequencing (NGS), T7 endonuclease I (T7E1) mismatch detection assay, and immunohistochemical staining (IHC) to confirm that the observed tumor growth suppression was attributable to the disruption of the RPS19 gene and the subsequent reduction in RPS19 protein expression. Evaluation of gene disruption efficiency via NGS demonstrated an impressive 56.2% efficiency for SNP_SS_(Cas9/sgRPS19), whereas no RPS19 cleavage was detected following treatment with normal saline or SNP_SS_(Cas9/sgCtrl) (Figure 6E). IHC also revealed a marked reduction in RPS19 expression in tumors treated with SNP_SS_(Cas9/sgRPS19). Editing of the RPS19 gene was confirmed by T7E1 assays, which revealed a mutation rate exceeding 50%, consistent with the results obtained from NGS (Figure 6F). Next, we evaluated changes in the TIME following nanoparticle treatments. The flow cytometry analysis revealed that the expression of cytotoxic T lymphocyte cytokines (TNFα, IFNγ, GZMB, and perforin) in the T cells of SNP_SS_(Cas9/sgRPS19) treated tumors was significantly higher than that in the other treatment groups (Figure 6G). Of greater importance, a decreased number of protumor TAMs was observed in tumor tissue samples from the SNP_SS_(Cas9/sgRPS19) treatment group, as evidenced by the downregulated expression of IL10 and CD206 (Figure 6H) and declined percentage of IL10^+^ and CD206^+^ macrophages (Figure 6I). In the orthotopic H22‐luc HCC model (Figure S14) and spontaneous HCC model (Figure S15), we repeatedly observed treatment with SNP_SS_(Cas9/sgRPS19) significantly reduced tumor progression, reversed inhibitory TIME and prolonged the survival of the mice. These results indicate that targeting RPS19 with SNP_SS_(Cas9/sgRPS19) in mice with orthotopic and spontaneous HCC can remodel the TME and effectively suppress HCC progression.

**FIGURE 6:**
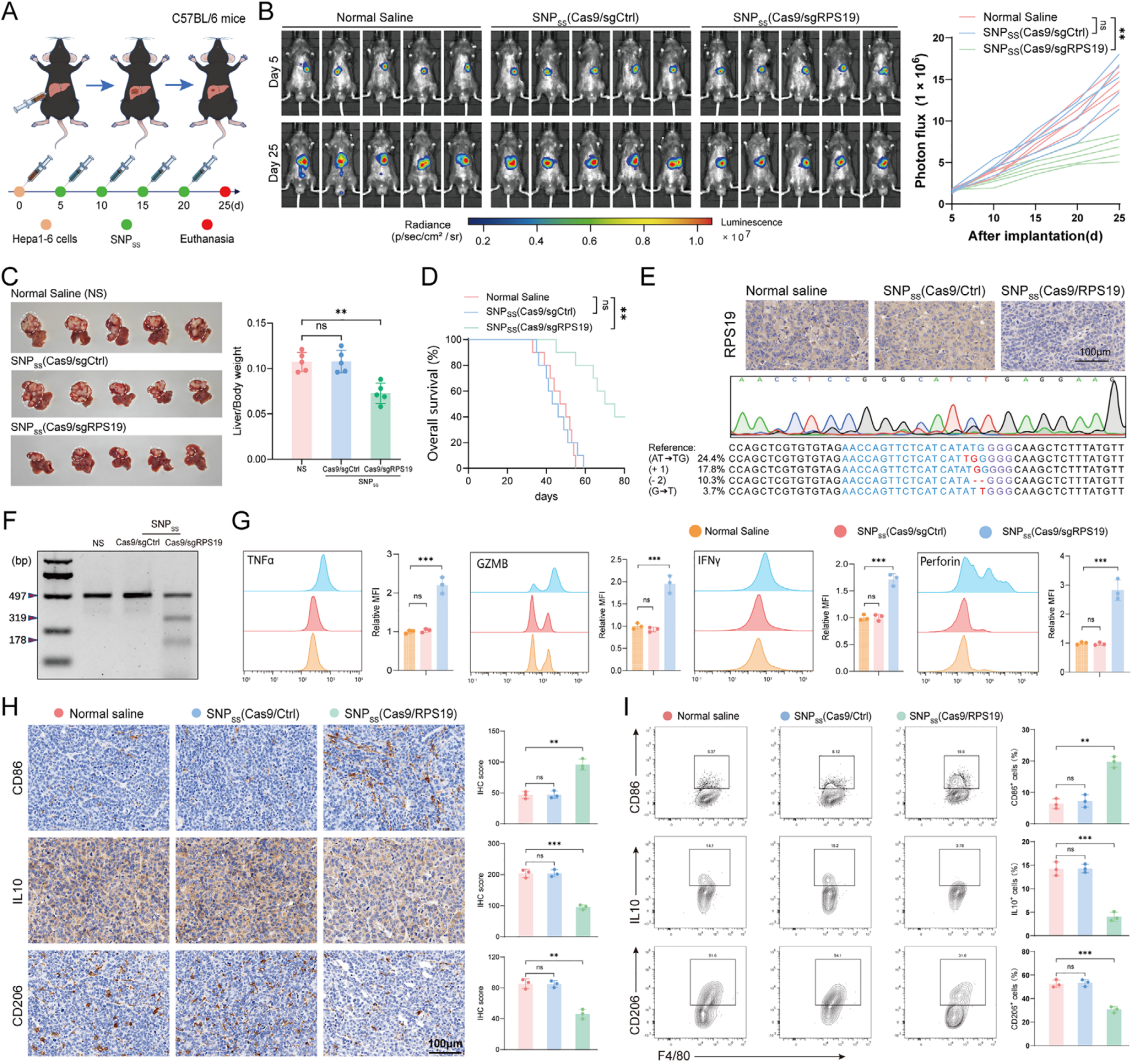
SNP_SS_(Cas9/sgRPS19) Nanoparticle‐Mediated Gene‐Editing Therapy Remodels the TIME and Effectively Suppresses HCC Progression in an Orthotopic Hepa1‐6‐luc HCC mouse model. (A) A diagram showing the timeline of the study performed using the Hepa1‐6 orthotopic tumor model. (B) Images showing luminescence in C57BL/6 mice bearing orthotopic Hepa1‐6‐Luc tumors at day 5 and day 25 post tumor inoculation (left), and changes in luminescence intensity of tumor regions over the course of treatment (right). (C) Photographs of liver tumors (tumor regions are demarcated by dotted lines) and liver‐to‐body weight ratios in mice following the indicated treatments on Day 25. (D) Three additional groups of mice were used to assess mouse survival (n = 10). (E) Results of IHC (up) and NGS (low) demonstrating RPS19 gene disruption in mouse tumors following the indicated treatments. (F) T7E1 assays demonstrating RPS19 gene disruption in mouse tumors following the indicated treatments. (G) Flow cytometry analysis showing the expression of TNFα, IFNγ, GZMB, and perforin in tumor‐infiltrating T cells of mice following the indicated treatments. (H) IHC analysis showing the expression of CD86, IL10, and CD206—markers of tumor‐associated macrophages (TAMs)—in mouse tumors following the indicated treatments. (I) Flow cytometry analysis showing the proportion of CD86, IL10, and CD206 positive macrophages in mouse tumors following the indicated treatments. *P* values were calculated using two‐way ANOVA (B), two‐tailed unpaired *t* tests (C, H and I) or log‐rank Mann–Cox tests (D). Data are presented as the means ± SDs (ns: not significant, **P < 0.01, and ***P < 0.001).

### 2.7 Genetic Inhibition of RPS19 Using SNP_SS_(Cas9/sgRPS19) Nanoparticles Synergistically Enhances Efficacy of Both anti‐PD‐1 and T+A Regimens in HCC

PD‐1/CTLA‐4 immunotherapy shows efficacy in multiple cancers [55], but the response rate of anti‐PD‐1 antibodies in HCC is below 20% [5]. Our study revealed that RPS19 promotes HCC progression by regulating the tumor immune microenvironment, suggesting that combined blockade of PD‐1 and RPS19 may improve anti‐tumor effects. We established an orthotopic HCC mouse model and treated mice with saline, SNP_SS_(Cas9/sgRPS19), anti‐PD‐1 antibody, or their combination. Notably, the combination therapy had greater efficacy than the other treatments, as evidenced by the strength of the bioluminescent signal (Figure 7A,B). Saline‐treated mice showed stronger bioluminescence and faster tumor growth. All three treated groups exhibited significantly smaller tumor volumes than the saline group, with the combination therapy group having the lowest tumor weight (Figure 7C). An analysis of survival curves indicated that the combination therapy increased the median survival to over 80 days, whereas the median survival durations for animals treated with SNP_SS_(Cas9/sgRPS19) and anti‐mPD1 alone were 68 and 73 days, respectively. In contrast, the median survival time of the saline‐treated group was markedly shorter, at only 44 days (Figure 7D). Furthermore, a T7E1 mismatch detection assay confirmed the disruption of the RPS19 gene in tumors from both the SNP_SS_(Cas9/sgRPS19) and combination treatment groups (Figure 7E). Multicolor immunofluorescence staining for DAPI, PanCK, CD8, GZMB, CD86, and CD206 was performed on tumor tissues from four mouse groups. Anti‐mPD1 and SNP_SS_(Cas9/sgRPS19) groups showed increased CD8, GZMB, CD86 and decreased CD206 expression, with more significant changes in the combination therapy group (Figure 7F,G). Using flow cytometry, we showed that the combination treatment, RPS19 gene editing, and PD1 antibodies significantly increased the expression of CD69, TNFα, and IFNγ in CD8^+^ TILs derived from Hepa1‐6 orthotopic HCC tissues. Importantly, the combination treatment outperformed monotherapy in activating CD8^+^ TILs (Figure 7H,J). Moreover, flow cytometry revealed the upregulation of CD86 and iNOS, coupled with the downregulation of CD206 and IL10, in F4/80^+^ macrophages following treatment. The effects of the combination therapy exceeded those of the individual drug treatments (Figure 7I,K). These findings confirm that genetic inhibition of RPS19 plus anti‐PD‐1 therapy effectively suppresses tumor growth, offering a potential strategy for immunosuppressive HCC.

**FIGURE 7:**
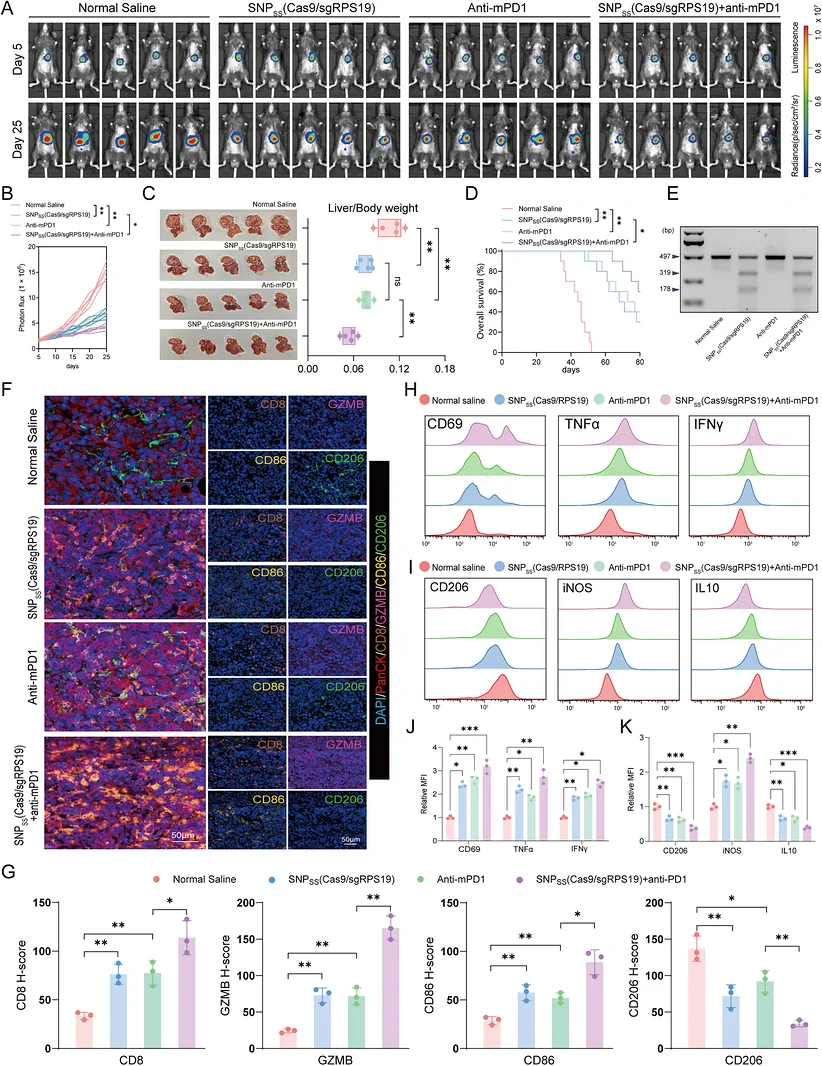
SNP_SS_(Cas9/sgRPS19) Nanoparticles Synergistically Enhances the Efficacy of Anti‐PD‐1 Antibody in an Orthotopic Hepa1‐6‐luc HCC mouse model. (A) Luminescence images of mice after treatment with normal saline, SNP_SS_(Cas9/sgRPS19), anti‐mPD1, or SNP_SS_(Cas9/sgRPS19) together with anti‐mPD1 (*n* = 5). (B) Luminescence intensity in the mice was measured via the IVIS Spectrum system following the indicated treatments. (C) Photographs of mouse livers and liver‐to‐body weight ratios in each group at Day 25 following the indicated treatments. (D) Mouse survival after indicated treatments was evaluated by using another three groups of mice (*n* = 10). (E) T7E1 assays demonstrating RPS19 gene disruption in mouse tumors following the indicated treatments. (F and G) Multiplex immunofluorescence staining demonstrating the expression levels of PanCK, CD8, GZMB, CD86, and CD206 in mouse tumor tissues at Day 25 following the indicated treatments. (H and J) Flow cytometry analysis and quantification of CD69+, TNFα+ and IFNγ+ T cells in mouse tumor tissues at Day 25 following the indicated treatments. (I and K) Flow cytometry analysis and quantification of iNOS+, CD206+ and IL10+ TAM cells in mouse tumor tissues at Day 25 following the indicated treatments. *P* values were calculated using two‐way ANOVA (B), two‐tailed unpaired *t* tests (C, G, J and K) and the log‐rank Mantel‒Cox test (D). Data are presented as the means ± SDs (ns: not significant, *P < 0.05, **P < 0.01 and ***P < 0.001).

Given the promising anti‐tumor effects of RPS19 editing observed in mouse HCC models, we next sought to validate its therapeutic potential, as well as its combinatorial benefit with immune checkpoint blockade, in a more clinically‐relevant human tumour system. We therefore performed functional assays using a humanized PBMC‐reconstituted HCC PDX model. NSG mice were reconstituted with human PBMCs isolated from five healthy donors and implanted with primary human HCC fragments, then randomly assigned to normal saline, SNP_SS_(Cas9/sgRPS19(h)), anti‐hPD‐1, or combination‐treatment groups (Figure S16A). While SNP_SS_(Cas9/sgRPS19) monotherapy slowed tumour growth and anti‐hPD‐1 alone showed modest efficacy, their combination exerted markedly stronger tumour‐suppressive effects (Figure S16B–D). Multiplex immunofluorescence showed reduced CD68^+^ macrophages, increased CD8^+^ T cells and higher GZMB levels upon RPS19 editing; these immune‐remodelling phenotypes were most prominent in the combination arm (Figure S16E). These findings confirm robust synergistic anti‐tumour activity of RPS19 editing together with anti‐hPD‐1 in human HCC PDX. We next assessed whether RPS19‐targeted genetic editing could synergize with the clinically relevant T+A regimen (anti‐mPD‐L1 plus BVZ) in an orthotopic Hepa1‐6 HCC model [56]. Tumour‐bearing mice were randomly assigned to four cohorts: Normal Saline, SNP_SS_(Cas9/sgRPS19) monotherapy, anti‐mPD‐L1 + BVZ dual therapy, and triple combination therapy combining SNP_SS_(Cas9/sgRPS19) with anti‐mPD‐L1 and BVZ (Figure S17A). Serial in vivo bioluminescence imaging tracked tumour burden throughout treatment, and liver‐to‐body weight ratios were quantified at experimental endpoint, the anti‐mPD‐L1 + BVZ dual regimen produced slightly stronger tumour‐growth inhibition than SNP_SS_(Cas9/sgRPS19) single treatment, the triple‐combination group exhibited the greatest anti‐tumour activity, significantly exceeding the efficacy of either monotherapy or dual‐agent therapy (Figure S17B,C). These results confirm that RPS19 editing synergizes with PD‐L1/VEGFA dual blockade, supporting the translational potential of combining RPS19 targeting with first‐line T+A‐based regimens for HCC.

TCGA pan‐cancer analysis showed RPS19 was upregulated in 27 tumor types versus normal tissues, with 11 showing significant differences (Figure S18A). Decreased survival times were associated with increased RPS19 expression across several tumor types, including ACC (adrenocortical carcinoma), KIRC (kidney renal clear cell carcinoma), KIRP (kidney renal papillary cell carcinoma), LGG (brain lower grade glioma), and SARC (sarcoma) (Figure S18B). The interrogation of TCGA data indicates that RPS19 holds potential as a therapeutic target in tumor types beyond HCC.

### 2.8 SNP_SS_(Cas9/sgRPS19) Delivery System has a Favorable Safety Profile

Our results confirm RPS19 as a key therapeutic target and validate our Cas9/sgRNA delivery system for efficient in vivo gene editing. Given safety concerns over toxicity and immunogenicity, we comprehensively evaluated the CRISPR/Cas9 nanoparticle system SNP_SS_(Cas9/sgRPS19). Deep sequencing in tumor‐bearing mice showed mutation frequencies below 0.5% at five potential off‐target sites in both tumors and major organs, indicating minimal off‐target risks (Figure S19A,B). To more precisely investigate the off‐target risk of the SNP_SS_(Cas9/sgRPS19) in vivo editing system beyond limited in‐silico prediction, we performed unbiased genome‐wide screening using the Site‐seq assay [57]. This assay identified the on‐target RPS19 site and top‐20 putative off‐target loci (Figure S19C). Subsequent targeted amplicon deep sequencing of mouse Hepa1‐6 tumour DNA confirmed 46% on‐target editing in the treatment group. By contrast, all 20 candidate off‐target sites exhibited indel frequencies <0.5%, with no obvious deviation from saline controls (Figure S19D). These data demonstrate minimal off‐target activity for SNP_SS_(Cas9/sgRPS19) in vivo.

In healthy Balb/c mice treated with a five‐fold therapeutic dose, histological analysis revealed no obvious damage to the heart, spleen, lungs, or kidneys (Figure S20A). Only mild liver injury with slightly elevated AST and ALP levels was detected, but no significant alterations in body weight, behavior, or four‐month survival were observed (Figure S20B–D). These findings support the good biosafety and tolerability of SNP_SS_(Cas9/sgRPS19) at therapeutic doses. The mild hepatic effects are likely associated with nanoparticle materials rather than CRISPR‐mediated off‐target mutations. Further comprehensive toxicological assessment is still needed for future preclinical translation.

## 3 Discussion

The liver possesses a well‐preserved immune system that collectively forms a robust network, supporting HCC amid its potent immunogenic properties and microbial environment [58]. Cytotoxic immune responses within the HCC tumor microenvironment are diminished primarily because of the proliferation of immunosuppressive cell types, including lymphocytes and myeloid cells, which promote tumor growth. Notably, immunotherapy has been shown to induce clinical remission in a large cohort of patients with advanced cancer [59]. Nonetheless, the low response rates present challenges for the effectiveness of immune checkpoint inhibitors (ICIs), including agents that target PD‐1, when they are administered to patients with advanced HCC [5, 60]. Identifying new targets to modulate the suppressive TIME in HCC is crucial for improving immunotherapy outcomes. In the current study, we constructed a mouse sgRNA library targeting over 4,500 genes associated with tumor initiation, progression, and immune modulation to identify potential immunotherapeutic targets involved in the complex crosstalk between the immune system and cancer cells. We also performed a customized in vivo CRISPR screen—accounting for variations in mouse immune responses—to detect alterations in immune‐related genes across diverse TIMEs implicated in immune surveillance and evasion. These targets may accelerate the development of innovative clinical interventions benefiting a substantial subset of patients with HCC. Overall, our study identified RPS19 as a key mediator of immune evasion. Genetic ablation of RPS19 in tumor cells enhances the susceptibility of HCC to ICB therapy, leading to complete or partial tumor regression and prolonged survival.

The gene encoding RPS19 is located on human chromosome 19q13.2 and plays a crucial role in the processing of pre‐rRNA and the maturation of the 40S ribosomal subunit [29, 61]. RPS19 is expressed in various tissues, including the intestine, nervous system, eyes, kidneys, liver, and muscles. Research indicates that RPS19 is overexpressed in bone marrow‐derived mesenchymal stem cells and that mutations affecting this gene are linked to Diamond–Blackfan anemia (DBA) [62]. Notably, elevated ribosomal protein levels are strongly associated with the occurrence of human cancers [63], with RPS19 being significantly overexpressed in tumor cells, suggesting the potential for malignancy [64, 65]. In the current study, we demonstrated a significant correlation between RPS19 expression and HCC progression across multiple HCC datasets. Furthermore, we confirmed that while RPS19 influences HCC cell growth, its modulation of the tumor microenvironment—specifically via suppression of anti‐tumor immune responses—represents a critical mechanism by which RPS19 promotes HCC progression. Previous studies have shown that RPS19, released from necrotic or apoptotic tumor cells, modulates the functions of various myeloid cells through the cell surface receptor C5aR1 [39, 40, 41]. Through comprehensive integrated analysis of published scRNA‐seq datasets and rigorous validation utilizing in vitro and in vivo experimental models, we identified macrophages as the predominant cell population responsible for mediating RPS19‐driven immunosuppression within the HCC microenvironment. In both human and murine macrophage populations, RPS19 transmits signals via C5aR1, which significantly upregulates the expression of protumorigenic mediators (e.g., VEGFA and TGFβ) as well as immunosuppressive factors that notably impair the cytotoxicity and chemotaxis of T cells (e.g., IL10 and CXCL9). These results establish a solid mechanism through which RPS19 exerts its immunosuppressive activity primarily by regulating the polarization of tumor‐associated macrophages; this, in turn, modulates the cytotoxic potential of T cells and ultimately drives the progression of HCC.

In the present study, we employed clodronate liposome‐mediated cell depletion to identify the cellular mediator of RPS19‐driven immune regulation. Nevertheless, clodronate liposomes lack strict cell‑type specificity for macrophage elimination, and multiple alternative strategies for TAM depletion have been established [49]. Macrophage‑targeted depletion approaches have been widely exploited to dissect TAM function and develop anticancer therapeutics. Pharmacological inhibition of CSF1R reduces intratumoral TAM abundance in preclinical models, yet yields limited clinical efficacy accompanied by dose‑limiting adverse effects such as ocular edema and hepatotoxicity [66]. CCL2‑CCR2 blockade represents another strategy to restrain monocyte recruitment into tumours; however, its clinical translation is hampered by rapid rebound of free CCL2 after treatment, which prevents sustained pathway suppression [67]. Moreover, genetically engineered transgenic models offer superior cell‑type specificity for TAM manipulation, though they cannot be readily translated to clinical application [68]. Accordingly, the optimal approach for TAM depletion should be carefully selected according to specific research objectives and translational scenarios.

Our study identified that RPS19 promotes HCC progression by suppressing anti‐tumor immunity in the tumor microenvironment. Disrupting RPS19 may remodel the immunosuppressive TIME, promoting anti‐tumoral macrophage polarization and T‐cell activation. However, the intracellular localization and physiological roles of RPS19 in normal tissues demand precise tumor‐targeted strategies. We applied gene editing to inactivate RPS19 in tumor cells. Current in vivo CRISPR‐Cas9 delivery systems have limitations: viral vectors trigger immune responses and off‐target effects [69], while nonviral systems suffer from low loading efficiency, poor specificity, and lack of responsive release [70]. Here, we established a glutathione‐responsive nanoparticle system to deliver CRISPR‐Cas9 ribonucleoprotein and sgRNA for RPS19 knockout in HCC [50]. The nanoparticles were functionalized with SP94 peptide, which specifically targets GPR78 on HCC cells, enabling tumor‐selective delivery [51]. Disulfide bonds in the nanoparticles respond to high glutathione levels in tumor cells, releasing the CRISPR payload precisely. In vivo studies showed efficient RPS19 knockout and significant anti‐tumor efficacy without obvious organ toxicity. This targeted nanoparticle‐mediated CRISPR system improves specificity and safety for RPS19‐targeted immunotherapy, providing a promising platform for in vivo gene‐editing cancer treatment.

In conclusion, we conducted an in vivo CRISPR screen and identified RPS19 as a critical factor in immune evasion, highlighting its potential as a target for enhancing HCC immunotherapy efficacy. We demonstrated that macrophages represent the critical cell population mediating the immunosuppressive functions of RPS19 in HCC, and that RPS19 induces an immunosuppressive phenotype in macrophages through binding to C5aR1. We developed a gene‐editing therapy utilizing nanoparticle‐mediated CRISPR/Cas9 to address HCC. By specifically knocking out RPS19 in HCC cells in a mouse model, we were able to reverse the immunosuppressive TME and promote the immune‐mediated destruction of tumor cells. This therapy, when combined with PD‐1 antibody immunotherapy, has synergistic effects on HCC.

## 4 Materials and Methods

### 4.1 Cell Culture

The H22, Hepa1‐6, THP‐1, and RAW264.7 cell lines used in this study were all obtained from the cell bank of our laboratory. These cell lines were originally purchased from the Cell Bank of the Chinese Academy of Sciences (Shanghai, China) and were cultured and cryopreserved strictly in accordance with the standard protocols recommended by the supplier. During in vitro culture, the cells were maintained in a humidified incubator with 5% CO_2_ at 37°C. Mycoplasma detection kits were routinely used to monitor and prevent mycoplasma contamination. To minimize the risks of cell phenotype drift, cellular senescence, and microbial contamination, regular cryopreservation was performed as a quality control measure. The cryopreserved cell aliquots were stored in liquid nitrogen at −196°C for long‐term preservation and subsequent experimental applications.

### 4.2 Mice

All animal experiments and related procedures in this study were performed in strict compliance with the protocol approved by the Animal Care and Use Committee of Fourth Military Medical University (no. IACUC‐230324). Mice were housed under standard environmental conditions, including a 12‐hour light/12‐hour dark cycle, and were provided with ad libitum access to sterile food and filtered water. For treatment‐related experiments, a randomized design was adopted, and drug injections were administered by researchers who were blinded to the group allocation of the experimental animals.

### 4.3 Establishment of Mouse Orthotopic HCC Model

Male C57BL/6J or Rag1^−/−^ mice (6–8 weeks old) were first anesthetized via intraperitoneal injection of 3% (w/v) sodium pentobarbital at a dose of 50 mg/kg (adjusted according to mouse weight to ensure stable anesthesia). Subsequently, 2×10^6^ Hepa1‐6 cells stably transfected with the luciferase gene were surgically implanted into the left lobe of the mouse liver using a sterile microsurgical technique. For Balb/c or nude mice, the orthotopic tumor modeling procedure was consistent with the method described above. Notably, when an equivalent number of luciferase‐expressing H22 cells was implanted, it resulted in significant ascites formation in the mice; therefore, the implantation dose was adjusted to 1×10^6^ cells per mouse for H22‐derived orthotopic HCC models. Tumor growth dynamics were non‐invasively monitored and quantified using the IVIS Lumina II in vivo bioluminescence imaging system at predetermined time points post‐implantation.

### 4.4 Establishment of Mouse Spontaneous HCC Model

Mouse spontaneous HCC model was conducted using the hydrodynamic tail vein injection (HTVI) technique, which entails the rapid administration of a large‐volume plasmid mixture into the mouse tail vein within a short time frame (typically 5–9 s). To establish the spontaneous HCC model, we performed HTVI of a plasmid mixture consisting of myr‐AKT1‐luc, N‐RasV12, and Sleeping Beauty transposase. Notably, we inserted a luciferase gene fragment into the myr‐AKT1‐encoding plasmid (#31789, Addgene) to generate the myr‐AKT1‐luc plasmid, which enabled real‐time monitoring of liver tumor size in mice using the IVIS Spectrum system. The injection mixture for each mouse was formulated as follows: 10 µg of the modified myr‐AKT1‐luc, 10 µg of N‐RasV12 (#20205, Addgene), 0.4 µg of Sleeping Beauty transposase (#24551, Addgene), and sterile normal saline to adjust the total volume to 2 mL. After plasmid injection, all mice were housed under standard environmental conditions, and tumor growth was dynamically monitored using the IVIS Lumina II in vivo imaging system.

### 4.5 In Vivo CRISPR Screening

Briefly, H22 cells were transduced with a lentivirus encoding the membrane‐bound artificial tumor antigen ovalbumin (OVA) (GL178, Obio‐tech) and cultured for 24 h. After blasticidin selection for 7 days, OVA expression levels of transduced cancer cells were identified by immunoblotting. The CRISPR library plasmids (#174197, Addgene) were transfected into HEK293FT cells at 90% confluence in 15 cm tissue culture plates. Viral supernatant was collected at 48 and 72 h post‐transfection, filtered via a 0.45 mm filtration unit (#430770, Corning). The sgRNA sequences encoded by the library plasmids and the names of targeted genes are provided in Table S1. For the pooled large‐scale CRISPR screen, more than 1×10^8^ OVA expressing H22 cells were transduced with the aforementioned lentiviral library at a multiplicity of infection (MOI) of 0.3. After a selection period of 3 days with puromycin, 30% of the remaining cells were utilized for subsequent in vitro or in vivo experiments. After selection, H22 cells transduced with the libraries were expanded in vitro or orthotopically implanted into Balb/c nude mice (immunodeficient), Balb/c mice (immunocompetent), or Balb/c + OVA mice (immunoenhanced). The Balb/c + OVA mice were vaccinated with ovalbumin peptide (#HY‐P0286, MCE) twice14 days before cancer cell transplantation (once a week). All mice were euthanized at 15 days post‐implantation, and tumor genomic DNA was extracted from whole tumor tissue using the HP Tissue DNA Midi Kit (Omega). The analysis of the sgRNA library involved a two‐step PCR process. In the first PCR step, a substantial amount of genomic DNA was utilized to ensure the preservation of the entire library complexity. In the second PCR step, specific sequencing adapters were subsequently added to the products obtained from the initial PCR amplification. Finally, the library for high‐throughput sequencing on the Illumina platform was obtained through purification. After the library is constructed, it undergoes an initial quantitative dilution using Qubit 3.0. The insert size and nucleic acid concentration of the library are detected using Agilent 2100. After pooling the samples, NanoQC is used to accurately quantify the effective concentration of the pooled library to ensure the accuracy of the instrument concentration and data output.

### 4.6 CRISPR Screen Data Analysis Pipeline

Following high‐throughput sequencing, raw sequencing data with adequate depth (≥200× coverage per guide RNA, a threshold ensuring reliable downstream analysis) were generated, which served as the starting point for bioinformatics analysis, and this analytical process primarily comprised three sequential stages: first, for gRNA sequence extraction and data quality control, sequencing reads containing gRNA sequences were extracted based on the library construction protocol, the core gRNA sequence was retained to generate gRNA‐specific FASTQ files, and subsequent quality control included statistical analysis of data output and assessment of sequence quality, with only samples meeting these quality criteria proceeding to downstream analysis; second, for gRNA alignment and read count quantification, quality‐passed reads were aligned to the reference gRNA library, key metrics evaluated post‐alignment included alignment rate, average sequencing depth per gRNA, and gRNA gene coverage, and further analysis was initiated only if all these metrics met pre‐established thresholds; third, for differential gRNA gene analysis between samples, aligned read counts were normalized using the trimmed mean of M‐values method to eliminate batch effects and sequencing depth bias, the MAGeCK (Model‐based Analysis of Genome‐wide CRISPR/Cas9 Knockout) software package was then employed to calculate fold changes in gRNA abundance between experimental and control groups, and statistical significance of differential gRNA gene expression was determined using the Benjamini‐Hochberg method.

### 4.7 Real‐Time Cell Analysis (RTCA) for Assessment of Cell Proliferation

Hepa1‐6_Ctrl (control) and Hepa1‐6_RPS19‐KO (RPS19 knockout) cells in the logarithmic growth phase were harvested and resuspended in sterile complete medium. Cell density was adjusted to 5×10^4^ cells/mL using a hemocytometer to ensure consistency across samples. Subsequently, 100 µL of each cell suspension was seeded into individual wells of an RTCA E‐Plate 96, with gentle shaking to ensure uniform cell distribution. The E‐Plate 96 was incubated in a humidified cell incubator at 37°C with 5% CO_2_ for 30 min to allow cell adhesion prior to RTCA monitoring. The xCELLigence RTCA system was programmed to automatically record the cell index (CI) every 10 min over a total duration of 96 h, thereby capturing real‐time cell proliferation dynamics. After the assay, raw data were exported, and proliferation curves were generated. Statistical analysis was performed to compare growth kinetics between the Ctrl and RPS19‐KO groups, focusing on differences in proliferation rate and peak cell index.

### 4.8 Acquisition and Analysis of Data From TCGA Database

The GEPIA online tool (http://gepia2.cancer‐pku.cn/#analysis) was employed to compare RPS19 expression levels between human cancer tissues and their paired adjacent normal tissues derived from the TCGA database. Additionally, this tool was used to explore the prognostic significance of RPS19 expression and its correlation with tumor grade across various human cancers. Subsequently, the preprocessed gene expression data (FPKM values) of hepatocellular carcinoma (TCGA‐LIHC cohort) was retrieved from the TCGA database using the Bioconductor TCGAbiolinks package (version 2.2.10). Gene set variation analysis (GSVA) was performed with the GSVA R package, wherein the gene sets were selected from the C2 collection (curated gene sets) downloaded from the MsigDB database (https://www.gsea‐msigdb.org/gsea/msigdb). Differences in pathway activities between clusters were analyzed using the Limma R package, and significantly dysregulated pathways were identified with a Benjamini‐Hochberg‐corrected P value < 0.01. Representative pathways associated with tumor progression, immune response, and immune regulation were selected for heatmap visualization.

### 4.9 Histology and Immunohistochemistry

The samples embedded in paraffin were sectioned sequentially, deparaffinized, and treated with 3% H_2_O_2_ to block endogenous peroxidase activity. The sections were then placed in antigen retrieval solution (10 mm sodium citrate acid, 0.05% Tween 20, pH 6.0) and heated to 120°C for 20 min, after which they were allowed to return to room temperature. Primary antibodies were applied, and the sections were incubated overnight at 4°C, after which secondary antibodies were added and incubated at room temperature for 1 h. Protein expression was quantified based on staining intensity and extent utilizing the histological scoring method described previously. In summary, the staining intensity was classified as follows: 0 for no staining, 1 for weak staining, 2 for moderate staining, and 3 for strong staining. The average proportion of stained cells per sample was assessed semiquantitatively. The IHC score for each sample was calculated as the product of the staining intensity and the percentage of positive tumor cells. An Olympus VS120 virtual slide scanning system was used to scan the tissue sections and tissue microarrays.

### 4.10 Western Blot

A protease inhibitor cocktail (Proteintech) was first diluted 1:100 in RIPA lysis buffer (Proteintech) and then added to the samples to prepare cell or tissue lysates. The protein concentration of each lysate was measured using the BCA assay and normalized to ensure consistency across samples. Proteins were subsequently separated via SDS‐PAGE and transferred onto PVDF membranes. The membrane was blocked with a 5% skim milk solution (BD) and incubated with primary antibodies overnight at 4°C, followed by an incubation with secondary antibodies at room temperature. After the chemiluminescent substrate was added, images were captured and analyzed using ImageJ software for densitometry of the immunoblot bands.

### 4.11 CyTOF and Data Analysis

Mass cytometry and data analysis were performed by Puluoting Health Tech Co., Ltd. (Hangzhou, China). Briefly, the cells were filtered through a 70‐µm cell strainer and subsequently treated with an anti‐mouse CD16/32 monoclonal antibody for 10 min at room temperature to inhibit Fc receptor activity. Each sample, containing 3×10^6^ cells, was then stained with a mixture of metal‐labeled monoclonal antibodies targeting specific cell surface markers, followed by processing with fixation/permeabilization buffer. The samples were subsequently incubated with a variety of monoclonal antibodies that recognize intracellular proteins. Finally, 1×10^6^ cells per sample were diluted in ddH2O with beads and analyzed using a mass cytometer (CyTOF, Fluidigm). All CyTOF files were normalized and manually gated in FlowJo software based on DNA content, event length, live/dead discrimination, CD45 expression, and 4 bead channels to eliminate dead cells, debris, doublets, nonimmune cells, and beads. The signal intensities (dual counts) per channel were arcsinh‐transformed with a cofactor of 5 (counts‐transf = asinh(x/5)). X‐shift clustering analysis was performed utilizing the R cytofkit package on all CD45^+^ cells from a total of 6 samples (pooled data) to automatically identify the underlying immune subsets. Heatmaps were generated based on the mean value for each marker in the clusters. The cell proportions within each cluster was calculated as the assigned cell events divided by the total number of CD45^+^ cell events in the same sample.

### 4.12 Acquisition and Analysis of scRNA‐seq Data

Normalized gene expression matrix data from single‐cell RNA sequencing (scRNA‐seq) of hepatocellular carcinoma (HCC) tissues were retrieved from the Genome Sequence Archive, which is hosted by the National Genomics Data Center (Beijing, China), under BioProject ID PRJCA007744. Subsequently, the “Seurat” package in R software was used to identify the major cell types within the HCC microenvironment. Cells expressing < 200 genes and genes expressed in fewer than 3 cells were excluded from further analysis. Initially, the read count for each cell was normalized through the following steps: dividing by the total read count of the respective cell, scaling the resulting value by a factor of 10 000, and subsequently applying a natural logarithm transformation. Hypervariable genes were identified using the “FindVariableFeatures” function, and these genes were used for principal component analysis (PCA) of the normalized gene expression matrix. The top 50 principal components (PCs) were selected for unsupervised clustering with a resolution of 0.5. Nearest neighbor graphs were constructed using the “FindNeighbors” function, and the uniform manifold approximation and projection (UMAP) algorithm was applied for nonlinear dimensionality reduction. Cluster‐specific marker genes were detected using the “FindAllmarkers” function in Seurat, with a threshold set at 0.25. Cell clusters were first annotated using the automatic cell type annotation R package “SingleR”, and the annotation results were further manually reviewed and adjusted based on the known canonical marker genes of each cell type. To investigate the differential composition of cell populations, the frequencies of major cell lineages were compared between groups. The Wilcoxon rank‐sum test was performed to analyze differences in cell type distribution frequencies. Additionally, the “FindMarkers” function in Seurat was used to detect differentially expressed genes (DEGs) between cohorts, with a threshold set at 0.25.

### 4.13 Inferring Copy Number Variations Based on scRNA‐seq Data

To effectively distinguish malignant from non‐malignant cells, large‐scale chromosomal copy number variations (CNVs) in single cells were inferred from scRNA‐seq data using the InferCNV tool (https://github.com/broadinstitute/inferCNV) with default parameters. Briefly, InferCNV first orders genes according to their genomic positions (chromosomes 1 to X, followed by gene start positions within each chromosome) and then employs a previously described sliding‐average strategy to normalize gene expression levels within fixed‐length genomic windows. Multiple putative non‐malignant cells were selected as references to further reduce noise in the CNV inference results. In the present analysis, endothelial cells and fibroblasts (hepatic stellate cells) were chosen as reference cell types to establish a baseline for CNV inference.

### 4.14 Cell‐Cell Communication Analysis

The cell‐cell communication analysis between tumor cells and immune cells was performed using the CellChat algorithm, with reference to the CellChatDB database. Cell type‐specific receptor‐ligand interactions were identified based on the specific expression pattern: receptors were expressed by one cell type, while ligands were expressed by another cell type. The magnitude of cell‐cell interaction was determined by the number and expression strength of receptor‐ligand pairs. Interactions among different cell subsets were visualized using the “ggplot2” R package.

### 4.15 CRISPR Knockout

Knockout cell lines were generated using lentivirus‐mediated CRISPR‐Cas9 technology. Specific sgRNAs targeting the mouse RPS19 gene were designed with an online tool developed by the F. Zhang laboratory at MIT in Boston, MA. The sgRNA sequences targeting the RPS19 gene were incorporated into the pLentiCRISPR‐v2 vector according to a standard cloning protocol. H22 and Hepa‐6 cells were infected with the lentivirus containing the sgRNA, followed by puromycin selection for three days to establish knockout lines. The surviving cells were subsequently plated in 96‐well plates via a limiting dilution strategy, and the resulting clones were expanded. The expression of the target protein was confirmed through western blot analysis.

### 4.16 TIF Collection

TIF was isolated from resected mouse engrafted HCC tumors following a well‐established mechanical centrifugation method. Briefly, freshly harvested tumor tissues were rinsed thoroughly with pre‐cooled sterile phosphate‐buffered saline (PBS) to remove residual blood, surface debris and tissue exudates. The cleaned tumor specimens were cut into small uniform fragments (approximately 3–5 mm in size) using sterile surgical scissors on ice. Subsequently, the tumor fragments were transferred onto 70 µm cell strainers pre‐installed in sterile 50 mL centrifuge tubes, allowing passive collection of interstitial fluid during centrifugation. The assembled samples were centrifuged at 400 × g for 15 min at 4°C to extract interstitial fluid from tumor tissues. After centrifugation, the crude TIF supernatant at the bottom of the centrifuge tube was carefully collected and further purified by filtration through a 0.22 µm sterile filter to remove cell debris, microvesicles and undigested tissue residues. The purified TIF samples were immediately snap‐frozen and stored at −80°C for subsequent ELISA quantification of soluble protein contents.

### 4.17 ELISA Binding and Competitive‐Binding Assays

Recombinant human C5aR1 protein was immobilized onto 96‐well microplates overnight at 4°C. After blocking with 5 % non‐fat milk in PBST for 2 h at room temperature, plates were washed three times with PBST. For saturation binding assays, serially diluted recombinant RPS19 or C5a (0–200 µM) was added to C5aR1‐coated wells, and incubated for 2 h at room temperature. Wells without C5aR1 coating served as negative controls. After washing, bound RPS19 and C5a were detected using corresponding primary antibodies, followed by HRP‐conjugated secondary antibodies. Optical density was measured at 450 nm. For reciprocal competitive‐binding ELISA, fixed concentration of RPS19 (200 µm) or C5a (200 µM) was incubated with immobilized C5aR1. Increasing concentrations of competing ligand were then supplemented. IgG was used as isotype control. After incubation and washing, bound ligand signals were quantified by OD450 reading as described above. For TIF ELISA quantification, TIF was isolated from orthotopic Hepa1‐6 tumor tissues of RPS19‐WT and RPS19‐KO mice. Soluble RPS19 and C5a protein concentrations in TIF were measured using commercial ELISA kits according to the manufacturer's instructions.

### 4.18 CCK‐8 Assay

Cells were seeded into 96‐well plates at a density of 3×10^3^ cells per well and cultured for 24, 48, 72, or 96 h. At each time point, 10 µL of CCK‐8 reagent (Beyotime) was added to each well, followed by incubation for 1–2 h at 37°C. Absorbance was measured at 450 nm using a microplate reader. For each condition, six replicate wells were measured, and the assay was independently repeated three times.

### 4.19 Isolation of Peripheral Blood Mononuclear Cells (PBMCs)

Peripheral blood samples were collected from the Department of Hematology, Xijing Hospital, into ethylenediaminetetraacetic acid (EDTA)‐anticoagulated tubes, following approval by the institutional ethics committee and obtaining written informed consent from all participants (no. KY20234106‐1). To maintain cell viability, samples were processed within 4 h of collection. First, whole blood was diluted at a 1:1 ratio with sterile phosphate‐buffered saline (PBS, pH 7.4) to reduce viscosity. A total of 10 mL of the diluted blood was carefully layered onto 5 mL of Ficoll‐Paque Plus (density: 1.077 g/mL) in a 15 mL centrifuge tube, with care taken to avoid disrupting the liquid interface. Centrifugation was performed at 400×g for 30 min at room temperature. The middle milky‐white PBMC layer was gently aspirated using a Pasteur pipette, transferred to a new centrifuge tube, and washed twice with PBS (centrifugation conditions: 300×g, 10 min, room temperature) to remove residual Ficoll‐Paque Plus and plasma components.

### 4.20 Polarization and Stimulation of Macrophages

Human macrophage cell line THP‐1 cells were cultured in RPMI‐1640 medium containing 20 ng/mL PMA for 48 h to induce differentiation into M0 macrophages. Subsequently, the medium was replaced with medium containing 1000 ng/mL LPS and 20 ng/mL IFN‐γ for 48 h to induce differentiation into M1‐type macrophages, or with medium containing 20 ng/mL IL‐13 and 20 ng/mL IL‐4 for 48 h to induce differentiation into M2‐type macrophages. Mouse macrophage cell line Raw264.7 cells were maintained in DMEM containing 10% FBS and 1% penicillin‐streptomycin. They were induced to differentiate into M1 type with 1000 ng/mL LPS and 20 ng/mL IFN‐γ, or into M2 type with 20 ng/mL IL‐13 and 20 ng/mL IL‐4. Human peripheral blood‐derived CD14^+^ monocytes were purified from PBMCs using CD14 immunomagnetic bead kits with 15 min incubation and magnetic column separation. For M0 polarization, sorted monocytes were resuspended in RPMI‐1640 medium supplemented with 50 ng/mL M‐CSF, seeded in plates, and cultured 6‐7 days. M1 macrophages were induced by treating mature M0 cells with 1000 ng/mL LPS and 20 ng/mL IFN‐γ for 48 h. M2 macrophages were induces 20 ng/mL IL‐4 and 20 ng/mL IL‐13 for 48 h. For studies on RPS19 protein, macrophages were co‐cultured with human or mouse RPS19 protein at different concentrations (5/10/20 ng/mL) for 48 h. For the C5AR1 antagonist PMX‐205, macrophages were pre‐incubated with it at a concentration of 1 µM for 5 h, followed by stimulation with RPS19 protein.

### 4.21 Humanized HCC PDX Model Construction

Fresh human HCC tumor specimens were obtained from patients undergoing surgical resection at Xijing Hospital with approved by the Ethics Committee of Xijing Hospital (no. KY20234106‐1). Tumor tissues were cut into approximately 3×3×3 mm fragments and subcutaneously implanted into the right flank of 6‑‐8‑week‑old female NSG mice. For human immune reconstitution, human peripheral blood mononuclear cells (PBMCs) were isolated from healthy‑donor buffy coats by Ficoll density‑gradient centrifugation, and 5×10^6^ PBMCs were intravenously injected into each NSG mouse 7 days prior to tumor implantation. When tumors reached approximately 100 mm^3^, mice were randomly assigned into four treatment groups (n = 5 per group): (1) Normal Saline vehicle control; (2) SNP_SS_(Cas9/sgRPS19) monotherapy, where sgRPS19 was designed to target the human RPS19 gene; (3) anti‑human PD‑1 antibody monotherapy; and (4) combination therapy of SNP_SS_(Cas9/sgRPS19) plus anti‑hPD‑1. SNP_SS_ nanoparticles were administered via tail‑vein injection every 5 days for a total of 4 doses, while anti‑hPD‑1 antibody was injected intraperitoneally every 3 days. Tumor volume and body weight were measured every 3 days. At the experimental endpoint, mice were euthanized, and tumors were harvested, weighed, and processed for downstream immunofluorescence analysis.

### 4.22 Quantitative Reverse Transcription Polymerase Chain Reaction (qRT‐PCR)

For qRT‐PCR analysis, total RNA was extracted from RPS19‐treated and untreated THP‐1 macrophages under M1 (LPS+IFN‐γ) or M2 (IL‐4+IL1‐3) polarization using TRIzol reagent. RNA concentration and purity were measured via a spectrophotometer. cDNA was synthesized from 1 µg RNA using a reverse transcription kit. qRT‐PCR was performed with gene‐specific primers for TNFα, INOS, CXCL9, IL10, CD209, TGFβ and GAPDH (housekeeping gene) on a real‐time PCR system. Reaction conditions: 95°C for 10 min, followed by 40 cycles of 95°C for 15 s and 60°C for 1 min. Relative gene expression was calculated using the 2^ (‐ΔΔCt) method, normalized to GAPDH.

### 4.23 Isolation of Multiple Human Primary Immune Cell Subsets

To isolate CD4^+^ T cells, CD8^+^ T cells, NK cells, B cells, and macrophages from human PBMCs, use immunomagnetic beads sorting: Resuspend PBMCs in separation buffer. For CD4^+^ T cells: Add anti‐human CD4‐conjugated beads (10 µL/1×10^6^ cells), incubate at 4°C for 20 min dark, load onto pre‐rinsed magnetic column, wash 3× with 500 µL buffer, elute with 1 mL buffer, centrifuge (300×g, 10 min). CD8^+^ T cells follow the same protocol with anti‐CD8 beads. For NK cells: Use anti‐CD56 beads; B cells: Anti‐CD19 beads; macrophages: Anti‐CD14 beads (all at 10 µL/1×10^6^ cells, same incubation/washing/elution steps). Post‐sorting, centrifuge all cell subsets to pellet. CD4^+^/CD8^+^ T cells can be TCR‐activated with anti‐CD3/CD28 antibodies in RPMI 1640 at 2×10^6^ cells/mL, cultured at 37°C 5% CO_2_ for 48 h. Macrophages require M‐CSF (50 ng/mL) in RPMI 1640 (10% FBS) for 6–7 days to differentiate into M0 phenotype.

### 4.24 Co‐Culture of CD8^+^ T Cells and Macrophages

Human peripheral blood mononuclear cells were differentiated into M0 macrophages with M‐CSF. CD8^+^ T cells isolated from the same source were TCR‐activated using anti‐CD3/anti‐CD28 beads with IL‐2 for 48 h. For co‐culture, M0 macrophages (1×10^5^/well) were seeded in 24‐well plates and allowed to adhere for 6 h. TCR‐activated CD8^+^ T cells were then added at a 2:1 ratio (T cells:macrophages); control wells contained only T cells. Part of the experimental groups received 10 nM recombinant RPS19 during co‐culture. All cultures were maintained in RPMI‐1640 at 37°C with 5% CO_2_ for 48 h. Suspended CD8^+^ T cells were collected, surface CD8, and intracellular Perforin, GZMB, and IFN‐γ, then analyzed by flow cytometry to quantify their expression.

### 4.25 Construction of Cas9/sgRPS19 Complex‐Encapsulating Nanoparticles

Positively charged Cas9 (20 µg) and negatively charged sgRPS19 (4.0 µg) were mixed via electrostatic adsorption in 500 µL of HEPES buffer (10 mm, pH 7.4) at a 1:1 molar ratio, followed by incubation at room temperature for 5 min to form the negatively charged Cas9/sgRPS19 complex. Subsequently, 5 µL of a positively charged acrylamide‐guanidine solution (1 mg/mL) was added to the reaction mixture, which was then vortexed at room temperature for 10 min to generate an electrically neutral nanoparticle core. Next, 44 µg of a neutral polyethylene glycol (PEG) derivative [acrylate polyethylene glycol succinimide carboxymethyl ester, bearing amide and succinyl functional groups] was added to the mixture, followed by vortexing at room temperature for 5 min. Thereafter, 10.4 µg of a degradable crosslinking agent [N,N'‐diblock(acryloyl)cystamine] was introduced into the system. To initiate polymerization, 3 µL of ammonium persulfate (catalyst, 1 mg/mL) and 3 µL of N,N,N',N'‐tetramethylethylenediamine (initiator, 1% v/v) were added, and the mixture was incubated at 4°C for 90 min. This reaction yielded nanoparticles encapsulating the Cas9/sgRPS19 complex. After nanoparticle formation, 90 µg of the SP94 peptide was added to the reaction system. The SP94 peptide was conjugated to the NHS ester groups at the terminal of PEG via an amide bond, thereby anchoring SP94 to the nanoparticle surface. The reaction product was diluted with standard PBS buffer (0.01 m, pH 7.4) and dialyzed using a nanodialysis device equipped with a polycarbonate membrane (pore size: 30 nm) to remove unbound RNA, unreacted proteins, and residual reaction components. Finally, trehalose was added as a lyoprotectant, and the mixture was freeze‐dried to obtain liver cancer‐targeted nanoparticles loaded with the Cas9/sgRPS19 complex.

### 4.26 RNase Protection Assay

We evaluated the stability of SNP_SS_(Cas9/sgRPS19) when exposed to RNase A. We incubated free Cas9/sgRPS19 or SNP_SS_(Cas9/sgRPS19) at a concentration of 200 nm in a solution containing RNase A (1 mg/ml) at 37°C for 30 min. Following this incubation, targeted DNA was added, and the mixture was further incubated at 37°C for 60 min to assess its ability to induce double‐strand breaks in the target DNA. Gel electrophoresis was then performed to analyze the extent of RNase protection.

### 4.27 T7E1 Assays and Sequencing

The T7E1 assay was employed to evaluate the efficacy of editing the targeted genomic loci. Cells or tissue samples were collected, and DNA was extracted using the DNA Isolation Kit from RiboBio. PCR amplification of each specific genomic locus was performed using the FastPure Gel DNA Extraction Mini Kit. Afterward, the standard T7E1 assay was executed. The disrupted segments on the gel were visualized using a gel documentation system and analyzed with ImageJ software. PCR products that exhibited mutations, as revealed by the T7E1 assay, were subsequently sent to Tsingke Biotech for Sanger sequencing. The same approach was utilized for in vivo gene editing and sequencing, with the exception that tumor genomic DNA samples were extracted using the Tissue DNA Kit from Omega, USA, after treatment with nanoparticles.

### 4.28 Antibody and Cytometry

Antibodies against mouse/human CD45, CD3, CD8, CD49b, TNFα, IFNγ, GZMB, perforin, CD69, F4/80, CD11b, CD86, CD206, CXCL9, iNOS, IL10, TGFβ and CD209 were purchased from eBioscience, BD, Thermo Fisher and BioLegend etc. For surface marker staining, 1×10^6^ collected cells from each group were resuspended in 50 µL of PBS containing 1 µl of fluorophore‐conjugated antibodies and incubated at room temperature in the dark for 30 min. The cells were then washed with 200–500 µL of PBS and analyzed with a CytoFLEX flow cytometer (Beckman, USA). For intracellular cytokine staining, the cells were stimulated with 20 ng/mL PMA (Sigma–Aldrich) and protein transport inhibitors (BD Biosciences) for 4 h prior to collection. The surface molecules were subsequently stained, and the cells were fixed with 100 µL of fixation/permeabilization solution (BD Biosciences) at room temperature for 15 min, followed by staining as described above.

### 4.29 Multiplex Immunofluorescence Staining

Tissue samples embedded in paraffin were sliced and stained using the OPAL multiplex fluorescence staining system provided by PerkinElmer. The sections were incubated at 62°C for 3 h, followed by deparaffinization using a Leica Bond RX. This step was succeeded by multiple rounds of primary antibody incubation and fluorescence coupling, adhering to the dilutions recommended by the manufacturer. After each primary antibody incubation, visualization was achieved through tyramide signal amplification with an Opal Polymer Horseradish peroxidase (HRP)‐conjugated secondary antibody, along with the following fluorophores: Opal 520, Opal 570, Opal 620, Opal 650, Opal 700, and DAPI (Akoya Biosciences). Protein expression was assessed based on the intensity and the positive staining percentage.

### 4.30 Safety Evaluation

To assess the safety of SNP_SS_(Cas9/sgRPS19), healthy Balb/c mice were used as subjects, with the formulation administered at a dose five times higher than that in antitumor experiments to address the propensity of nanoparticles to accumulate in major organs (heart, liver, spleen, lungs, and kidneys). A saline‐treated group served as the control. The mice received intravenous injections every five days, totaling four treatments. Post‐administration, histological examinations were conducted on the five target organs to evaluate tissue damage and inflammatory responses. For liver function assessment, we analyzed the concentrations of albumin (ALB), aspartate aminotransferase (AST), alanine aminotransferase (ALT), and alkaline phosphatase (ALP) in the blood using the services provided by Wuhan Servicebio Technology. Additionally, the mice were monitored for changes in body weight and food intake throughout the study, and their survival rates were tracked for four months to assess long‐term systemic effects.

### 4.31 Detection of Off‐Target Effects by Deep Sequencing

To evaluate the off‐target effects of nanoparticles, we predicted potential off‐target sites using the online database (https://cm.jefferson.edu/Off‐Spotter/) based on the following established criteria: (i) Cas9 exhibits greater tolerance to single‐base mismatches in the protospacer adjacent motif (PAM)‐distal region compared to the PAM‐proximal region; (ii) three or more mismatched base pairs eliminate detectable Cas9 cleavage in the vast majority of loci. Briefly, after treatment with SNP_SS_(Cas9/sgRPS19), genomic DNA was extracted from tumor tissues, normal tissues of mice bearing Hepa1‐6 using the Universal Genomic DNA Kit (CWBIO, China) following the manufacturer's instructions. One hundred nanograms of genomic DNA was used as a template for PCR amplification with primers designed to target both on‐target and off‐target sites. The purified DNA was subjected to a second PCR amplification with primers containing sequencing adapters, followed by sequencing and analysis by Sangon Biotechnology Company (Shanghai, China) to detect indels flanking the target sites.

### 4.32 Site‐seq

Unbiased genome‐wide CRISPR‐Cas9 off‐target profiling was performed using Site‐seq. Briefly, genomic DNA was incubated with pre‐assembled Cas9‐sgRPS19 RNP complex (sgRNA sequence: 5′‐AACCAGTTCTCATCATATG‐3′) for in vitro DNA cleavage. The reaction was terminated, and cleaved DNA fragments were purified for library construction, including end‐processing, sequential adapter ligation, magnetic‐bead purification and PCR enrichment, followed by high‐throughput sequencing. Bioinformatic analysis: Raw reads were quality‐filtered with cutadapt; PCR duplicates were removed via UMI correction. Clean reads were mapped to mouse reference genome using BWA. Candidate cleavage sites were identified by the Site‐seq pipeline, and genomic‐region annotation was conducted with ANNOVAR. The top‐20 high‐risk candidate off‐target loci identified by Site‐seq were selected for subsequent targeted amplicon deep‐sequencing validation in vivo.

### 4.33 Statistical Analyses

For each experiment, the figure legends detail the number of biological replicates and the statistical analyses conducted. A two‐tailed t test was employed for comparisons between two groups; for comparisons involving three or more groups, ANOVA was performed, followed by either Dunnett's test or Tukey's post hoc test. The survival analysis was executed using the log‐rank test, with the Bonferroni adjustments applied for multiple comparisons. All statistical graphs were generated using GraphPad Prism 10.0 (GraphPad Software, Inc., San Diego, CA), and figure assembly and illustration preparation were performed using Adobe Illustrator 2021 (Adobe Inc.).

### 4.34 Declaration of AI Use

During the preparation of this manuscript, the authors used the AI‐based language tool DeepSeek solely for language polishing and grammar refinement of the text. No AI tools or AI‐assisted technologies were used to generate, modify, enhance, or analyze any figures, images, graphical abstracts, or other visual content, nor were AI tools used for data analysis, experimental design, or the interpretation of scientific results. All scientific content and conclusions are entirely the work of the authors.

## Author Contributions

ML, HD, JG and KZ designed the study, interpreted data and wrote the manuscript. LH and DY conducted the tumor models and in vivo experiments. FX and KH conducted the in vitro experiments. ZL, MD and JY conducted the western blot analysis and immunoprecipitation experiments. HC, YX, YQ and GS helped with the design of experiments and coordinated the study. WZ, SW and QH contributed key reagents. JZ, ZW and YZ led liver cancer clinical team/core that provided samples for analysis.

## Funding

This work was supported by Innovative Drug Research and Development National Science and Technology Major Project (Grant No. 2026ZD1805800), the National Natural Science Foundation of China (82674277 and 82607674), the Program of the State Key Laloratory of Holistic Integrative Management of Gastrointestinal Cancers (2025GTKP008, 2025GTEP016, 2025GTEP017), and the Tangdu Hospital 2024 Discipline Promotion Plan (2024JCRH032).

## Ethics Statement

This study has been reviewed and approved by the Ethics Committee of Xijing Hospital of the Fourth Military Medical University (no. KY20234106‐1). All research involving human participants was conducted in accordance with both the Declaration of Helsinki. Written informed consent was obtained from all patients and healthy donors prior to sample collection. All animal experiments and related procedures in this study were performed in strict compliance with the protocol approved by the Animal Care and Use Committee of the Fourth Military Medical University (no. IACUC‐230324).

## Conflicts of Interest

The authors declare no conflicts of interest.

## Supporting information




**Supporting File 1**: advs78430‐sup‐0001‐SuppMat.docx.

**Supporting File 2**: advs78430‐sup‐0002‐TableS1.xlsx.

## Data Availability

The data that support the findings of this study are available from the corresponding author upon reasonable request.
