# Supplementary material for: In Vivo CRISPR Screen Identifies RPS19 as an Immunotherapy Target in Hepatocellular Carcinoma
Source: Adv Sci (Weinh). 2026 Oct 25:e78430. Online ahead of print. doi: 10.1002/advs.78430 (PMC13696879; doi:10.1002/advs.78430)
Supplement: Supplementary file 1 [file ADVS-9999-e78430-s001.docx]

**Supplementary Materials for**

**In Vivo CRISPR Screen Identifies RPS19 as an Immunotherapy Target in Hepatocellular Carcinoma**

**Authors:** Lei He^1*^, Duo Yu^1,3*^, Fei Xie^1*^, Kaisheng Hou^1*^, Zhaozhao Li^1^, Meifang Dai^1^, Jingyi Yun^1^, Haiyan Cao^1,4^, Yang Xiao^1,5^, Yueyuan Qiu^1^, Gege Shi^1^, Wangqian Zhang^1^, Shuning Wang^1^, Qiang Hao^1^, Jieyu Zhang^1^, Zhaowei Wang^1^, Yingqi Zhang^1^, Kuo Zhang^1#^, Jintao Gu^1#^, Haichen Du^2#^ and Meng Li^1#^.

^1^State Key Laboratory of Holistic Integrative Management of Gastrointestinal Cancers, Department of Biopharmaceutics, School of Pharmacy, Fourth Military Medical University, Xi’an, China

^2^Department of Oncology, 940th Hospital, Joint Logistic Support Force, Lanzhou, China

^3^Department of Neurosurgery, General Hospital of Central Theater Command, Wuhan, China

^4^Department of Neurosurgery, Tangdu Hospital, Fourth Military Medical University, Xi’an, China

^5^Department of Experiment Surgery, Xijing Hospital, Fourth Military Medical University, Xi’an, China

^*^These authors contributed equally to this work

^#^Corresponding author

Meng Li, MD., State Key Laboratory of Holistic Integrative Management of Gastrointestinal Cancers, Department of Biopharmaceutics, School of Pharmacy, Fourth Military Medical University, Xi’an, China (limeng@fmmu.edu.cn)

Haichen Du, MD., Department of Oncology, 940th Hospital, Joint Logistic Support Force, Lanzhou, China (DHC376741013@outlook.com)

Jintao Gu, MD., State Key Laboratory of Holistic Integrative Management of Gastrointestinal Cancers, Department of Biopharmaceutics, School of Pharmacy, Fourth Military Medical University, Xi’an, China (gujintao@fmmu.edu.cn)

Kuo Zhang, MD., State Key Laboratory of Holistic Integrative Management of Gastrointestinal Cancers, Department of Biopharmaceutics, School of Pharmacy, Fourth Military Medical University, Xi’an, China (justy1985@126.com)

**SUPPLEMENTARY FIGURE 1-20**

**Supplementary Figure 1. An In Vivo CRISPR Screen Identifies RPS19 as a Pivotal Immune Evasion Driver.**

**(A)** Pie chart showing the fraction of genes targeted in the MusCK library. **(B)** Protein Cas9 and Ovalbumin expression in MusCK library-infected H22 cells. **(C)** A matrix of the Pearson’s correlations of the library distribution from one condition compared to any other condition for MusCK screen. **(D)** High RPS19 expression correlates with poorer survival in Atezolizumab + Bevacizumab treated HCC patients. **(E)** RPS19 expression was evaluated by IHC scores in 40 HCC tissues, stratified according to WHO histological grades I–IV. Scale bars: 100 μm. **(F)** RPS19 expression, evaluated by Western blot, is shown in representative HCC tissues stratified according to WHO histological grades I–IV. *P* values were calculated using log‑rank test **(D)** and two-tailed unpaired *t* tests **(E)**. Data are presented as the means ± SDs (**P* < 0.05 and ***P* < 0.01).

**Supplementary Figure 2. RPS19 Mediated Immunosuppression Plays a Critical Role in HCC Progression.**

**(A and B)** Ki67, CD8 and CD206 expression, evaluated by IHC scores, is shown in representative HCC tissues stratified according to RPS19 expression (n=40, Scale bars: 100 μm). **(C)** RPS19 expression in monoclonalized RPS19-knockout Hepa1-6 and H22 cells. **(D)** The sanger sequencing validation of RPS19 knocking out in monoclonalized Hepa1-6 and H22 cells.  **(E)** The in vitro growth of RPS19 knock out (RPS19KO) and control knock out (Ctrl) Hepa1-6 mouse hepatoma cells monitored by Agilent xCELLigence real-time cell analysis (RTCA). **(F)** The in vitro growth of RPS19 knock out (RPS19KO) and control knock out (Ctrl) Hepa1-6, H22 mouse hepatoma cells monitored by CCK8 assay. **(G and I)** The Hepa1-6_RPS19KO and Hepa1-6_Ctrl cell growth in the livers of Rag1^-/-^ mice monitored by an IVIS Lumina II imaging system. **(H and J)** The Hepa1-6_RPS19KO and Hepa1-6_Ctrl cell growth in the livers of C57BL/6 wildtype mice monitored by an IVIS Lumina II imaging system. **(K)** The tumor luminescent signals in Rag1^-/-^ mice and wild-type mice bearing orthotopic Hepa1-6-Luc liver tumors on Days 20 following cell implantation. **(L)** The growth disparity between Hepa1-6_RPS19KO and Hepa1-6_Ctrl cells in Rag1^-/-^ mice and C57BL/6 mice. *P* values were calculated using two-tailed unpaired t tests **(B, F, K and L)** and two-way ANOVA **(E, I and J)**. Data are presented as the means ± SDs (ns: not significant, **P* < 0.05, ***P* < 0.01 and ****P* < 0.001).

**Supplementary Figure 3. The single-cell mass cytometry (CyTOF) analysis of tumor tissues derived from RPS19 knock out and control knock out H22 cells inoculated wildtype Balb/c mice.**  *t-*SNE plots illustrating the frequency of cells positive for T-cell activation and cytotoxicity markers (CD69, IL2, TNFα), NK cell markers (CD49b), M1 macrophage markers (CD86, iNOS), and MDSC markers (CD11b) within CD45^+^ tumor-infiltrating immune cells from orthotopic H22_RPS19KO or H22_Ctrl tumors in Balb/c mice. *P* values were calculated using two-tailed unpaired *t* tests (n=3). Data are presented as the means ± SDs (**P* < 0.05 and ***P* < 0.01).

**Supplementary Figure 4. Flow cytometry analysis the proportion of multiple immune cells in each group.**

**(A)** Representative contour plots and percentages of GZMB^+^ CD8^+^ T cells and Perforin^+^ CD8^+^ T cells in the RPS19-KO group and control group analyzed by flow cytometry. **(B)** Representative contour plots and percentages of IFN-γ^+^ CD49b^+^ NK cells and TNF-α^+^ CD49b^+^ NK cells in the RPS19-KO group and control group analyzed by flow cytometry. **(C)** Representative contour plots and percentages of Gr-1^+^ CD11b^+^ MDSC cells and Ly6G^+^ Gr-1^+^ MDSC cells in the RPS19-KO group and control group analyzed by flow cytometry. **(D)** Representative contour plots and percentages of CD86^+^ F4/80^+^ Macrophages and CD206^+^ F4/80^+^ Macrophages in the RPS19-KO group and control group analyzed by flow cytometry. *P* values were calculated using two-tailed unpaired *t* tests (*n*=5) **(A-D)**. Data are presented as the means ± SDs (***P* < 0.01 and ****P* < 0.001).

**Supplementary Figure 5.** **scRNA-seq Analysis of the Datasets from Genome Sequence Archive at the National Genomics Data Center (Beijing,China) under the BioProject ID PRJCA007744.**

**(A)** Heatmap presenting the marker genes of the annotated nine cell subsets. NK, natural killer cells; DCs, dendritic cells; MDSCs, myeloid-derived suppressor cells; HSCs, hepatic stellate cells. **(B)** Copy number variations (CNV) analysis profiles of the annotated hepatocytes. HSCs and endothelial cells are used as references. **(C)** Global intercellular signaling analysis with cell types scored based on their outgoing and incoming contributions to the network.

**Supplementary Figure 6. Multiplex immunofluorescence confirms spatial co‑localization of RPS19 and C5aR1 in mouse and human HCC.**

**(A)** Orthotopic Hepa1‑6 mouse HCC tissue. Left: low‑power merged view (200 μm scale bar). Zoom‑in images (80 μm scale bar) display merged channels, DAPI (blue), C5aR1 (yellow), RPS19 (purple). Fluorescence line‑intensity analysis shows overlapping distribution. **(B)** Human HCC tissue sections with the same staining setup.

**Supplementary Figure 7. The RPS19‑C5aR1 axis preferentially targets the Mac_0 immunosuppressive TAM sub‑cluster in HCC.**

**(A)** Dot plot showing RPS19 expression across major TME cell types in IA (Immune Activation) and IE (Immune Exclusion) groups. **(B)** UMAP of re‑clustered tumor‑associated macrophages, depicting five transcriptionally distinct TAM sub‑clusters (Mac_0-Mac_4). **(C)** Split UMAP projections of TAMs stratified by IA and IE groups. **(D)** Stacked‑bar plot illustrating TAM sub‑cluster proportions in IA versus IE. **(E)** Violin plot of C5AR1 transcript levels in Mac_0, C5AR1 is significantly higher in IE (RPS19‑high) Mac_0. **(F)** Violin plots for pro‑inflammatory (TNF, CXCL9) and immunosuppressive (TGFB1, CD209) genes in Mac_0. **(G‑H)** CellChat analysis of hepatocyte‑macrophage ligand‑receptor crosstalk. *P* values were calculated using two-tailed unpaired t tests **(E-F)**. Data are presented as the means ± SDs.

**Supplementary Figure 8. RPS19 release originates from dying tumor cells instead of exosomal secretion.**

**(A**) ELISA measurement of soluble RPS19 in culture supernatants from monocultured Hepa1‑6(RPS19‑WT), Hepa1‑6(RPS19‑KO), CD8⁺ T cells, and their corresponding co‑cultures (E:T = 10:1, 48 h). **(B)** LDH release assay assessing tumor cell lysis in the above‑mentioned culture systems. **(C)** ELISA detection of RPS19 in exosome‑depleted supernatant after ultracentrifugation. **(D)** ELISA analysis of RPS19 in RIPA‑lysed exosome pellets isolated from culture supernatants. *P* values were calculated using two-tailed unpaired *t* tests (*n*=5). Data are presented as the means ± SDs (ns: no significance; ****P* < 0.001).

**Supplementary Figure 9. *In‑vitro* binding and competition assays, together with intratumoral ligand quantification.**

**(A-B)** ELISA showing dose‑dependent binding of RPS19 and C5a to C5aR1. **(C-D)** Reciprocal competitive ELISA demonstrating mutual displacement between RPS19 and C5a for C5aR1 binding. **(E)** Quantification of soluble RPS19 and C5a in orthotopic Hepa1‑6 tumor lysates. *P* values were calculated using two-tailed unpaired *t* tests **(A, B and E)**. Data are presented as the means ± SDs (ns: no significance; ****P* < 0.001).

**Supplementary Figure 10. RPS19 regulate the expression of multiple macrophage relative markers.**

**(A)** Relative mRNA expression of CXCL9, TNF and INOS in THP-1 macrophages treated with varying concentrations of RPS19. **(B)** Relative mRNA expression of IL10, TGFβ and CD209 in THP-1 macrophages treated with different concentrations of RPS19. **(C)** Flow cytometry plot depicting the gating strategy for human peripheral blood macrophages, identified by the co-expression of CD14 and CD68. **(D-E)** Flow cytometry histograms (left) and quantification (right) of CXCL9, TNFα, CD209, TGFβ and IL10 expression (mean fluorescence intensity, MFI) in human peripheral blood macrophages treated with different concentrations of RPS19. **(F)** Flow cytometry histograms (left) and quantification (right) of CXCL9, TNFα, CD209, TGFβ and IL10 expression (MFI) in mouse Raw264.7 macrophages treated with different concentrations of RPS19. **(G)** Flow cytometry plot depicting the gating strategy for human peripheral blood, identified by CD3 and CD8 expression. *P* values were calculated using two-tailed unpaired *t* tests (*n*=3) **(A-B and D-F)**. Data are presented as the means ± SDs (**P* < 0.05, ***P* < 0.01 and ****P* < 0.001).

**Supplementary Figure 11. Expression of C5AR1 in human peripheral blood immune cells and macrophage cell lines.**

**(A-B)** Flow cytometry plots showing the gating strategy to identify C5AR1⁺ populations in human peripheral blood immune cells, including CD4⁺ T cells, CD8⁺ T cells, NK cells, B cells, and macrophages. The percentage of C5AR1⁺ cells within each gated population. **(C-D)** Flow cytometry plots showing C5AR1 expression in mouse macrophage cell line Raw264.7, human macrophage cell line THP1 and C5AR1 knockout THP1 cells (THP1-C5AR1KO). The percentage of C5AR1⁺ cells within each gated population. *P* values were calculated using two-tailed unpaired *t* tests (*n*=3) **(A and B)**. Data are presented as the means ± SDs.

**Supplementary Figure 12. Regulation of THP-1 and PBDM relative markers expression by RPS19 and PMX205.**

**(A)** Flow cytometry histograms (left) and quantification (right) of CXCL9, TNFα, CD209, TGFβ, IL10 expression (MFI) in THP1 cells treated with RPS19 or RPS19+PMX205. **(B)** Flow cytometry histograms (left) and quantification (right) of CXCL9, TNFα, CD209, TGFβ, IL10 expression (MFI) in primary human peripheral blood-derived macrophages (PBDM) treated with RPS19 or RPS19+PMX205. *P* values were calculated using two-tailed unpaired *t* tests (*n*=3) **(A and B)**. Data are presented as the means ± SDs (***P* < 0.01 and ****P* < 0.001).

**Supplementary Figure 13. Targeting of nanoparticles in spontaneous HCC model.**

**(A)** Fluorescence images of spontaneous HCC model mice following injection of Cy5.5 labeled NP_SS_(Cas9/sgRPS19) or SNP_SS_(Cas9/sgRPS19). Tumor regions are indicated by luminescence. **(B)** Time-dependent changes in Cy5.5 fluorescence within liver regions of mice administered NP_SS_(Cas9/sgRPS19) or SNP_SS_(Cas9/sgRPS19). **(C)** Measurements of luciferase luminescence and Cy5.5-Cas9 fluorescence in the heart, liver, spleen, lung, and kidney of C57BL/6 mice with spontaneous HCC model at 24 hours following intravenous injection of NP_SS_(Cas9/sgRPS19) or SNP_SS_(Cas9/sgRPS19). Tumor regions are demarcated by dotted lines. *P* values were calculated using two-tailed unpaired *t* tests **(A and C)** or two-way ANOVA **(B)**. Data are presented as the means ± SDs (****P* < 0.001).

**Supplementary Figure 14. SNP_SS_(Cas9/sgRPS19) Nanoparticle-Mediated Gene-Editing Therapy in a H22-luc Orthotopic HCC Mouse Model.**

**(A)** Diagram illustrating the timeline of the study conducted using the H22 orthotopic tumor model. Intravenous injection was performed on Days 5, 10, 15 and 20 after tumor implantation. Mice were euthanized on Day 25 post-tumor implantation (*n* = 5). **(B)** Images displaying luminescence in orthotopic H22-Luc tumor-bearing Balb/c mice following indicated treatments. **(C)** Quantification of luminescence levels in mice utilizing the IVIS spectrum system after indicated treatments. **(D)** Comparison of liver weight to body weight ratio among the groups following indicated treatments on Day 25. **(E)** Mouse survival after indicated treatments was evaluated by using another three groups of mice (*n* = 10). **(F)** The results of T7E1 cleavage assays displaying RPS19 gene editing in tumors. **(G)** IHC analysis of RPS19, CD8, Ki67, CD86, IL10 and CD206 expression in tumor tissues excised from mice receiving indicated nanoparticles. *P* values were determined by two-way ANOVA **(C),** two-tailed unpaired t test **(D and G)** and Mantel-Cox test **(E).** Data are presented as the means ± SD (ns: no significance; ***P* < 0.01).

**Supplementary Figure 15. SNP_SS_(Cas9/sgRPS19) Nanoparticle-Mediated Gene-Editing Therapy in a Spontaneous HCC Mouse Model.**

**(A)** Diagram illustrating how the spontaneous HCC model was established through a transposon system and the timeline of the nanoparticles treatment. Intravenous injection was performed on Days 5, 10, 15 and 20 after tumor implantation. Mice were euthanized on Day 25 post-tumor implantation (*n* = 5). **(B)** Images displaying luminescence in spontaneous HCC C57BL/6 mice and the quantification of luminescence levels in mice utilizing the IVIS spectrum system following indicated treatments. **(C)** Photographs of mouse livers, liver-to-body weight ratios, and counts of liver nodules in each group at Day 25 following the indicated treatments. **(D)** The results of T7E1 cleavage assays displaying RPS19 gene editing in tumors. **(E)** Mouse survival after indicated treatments was evaluated by using another three groups of mice (*n* = 10). **(F)** IHC analysis of RPS19, CD8, Ki67, CD86, IL10 and CD206 expression in tumor tissues excised from mice receiving indicated nanoparticles. *P* values were determined by two-way ANOVA **(B),** two-tailed unpaired t test **(C and F)** and Mantel-Cox test **(E).** Data are presented as the means ± SD (ns: no significance; ***P* < 0.01 and ****P* < 0.001).

**Supplementary Figure 16. Combination therapeutic effect of SNP_SS_ (Cas9/sgRPS19) and anti‑hPD‑1 in humanized HCC PDX mice.**

**(A)** Workflow diagram showing humanized PDX model construction and drug‑administration regimen. Human PBMCs were reconstituted in NSG mice before engraftment of patient‑derived HCC fragments. **(B)** Tumor‑volume growth curves for four treatment groups (Normal Saline, SNP_SS_(Cas9/sgRPS19), Anti‑hPD1, combination; n=5). **(C)** Tumor‑weight quantification at termination. **(D)** Representative photographs of resected PDX tumors. Scale bar=1 cm. **(E)** Multiplex immunofluorescence for DAPI, CD68, CD8 and GZMB in tumor sections. *P* values were determined by two-tailed unpaired t test **(C).** Data are presented as the means ± SD (ns: no significance; ***P* < 0.01 and ****P* < 0.001).

**Supplementary Figure 17.** **RPS19 editing synergizes with anti‑mPD‑L1 plus BVZ therapy in orthotopic HCC.**

**(A)** Representative in‑vivo bioluminescence images of orthotopic Hepa1‑6 HCC‑bearing mice across four experimental groups. **(B)** Luminescence intensity in the mice was measured via the IVIS Spectrum system following the indicated treatments. **(C)** Quantitative analysis of liver‑to‑body weight ratio at experimental endpoint. *P* values were determined by two-tailed unpaired t test **(C).** Data are presented as the means ± SD (**P* < 0.05; ***P* < 0.01 and ****P* < 0.001).

**Supplementary Figure 18. The expression of RPS19 across pan-cancers.**

**(A)** The expression of RPS19 across pan-cancers and their corresponding normal tissues in TCGA database. **(B)** Kaplan-Meier plot illustrating the survival of patients with various cancers from the TCGA database, stratified by high versus low RPS19 expression (Cox proportional hazards test). ACC (Adrenocortical carcinoma), KIRC (Kidney renal clear cell carcinoma), KIRP (Kidney renal papillary cell carcinoma), LGG (Brain Lower Grade Glioma) and SARC (Sarcoma).

**Supplementary Figure 19. Genome‑wide specificity evaluation of in‑vivo SNP_SS_(Cas9/sgRPS19) editing.**

**(A-B)** Orthotopic Hepa1-6 HCC mouse model treated with SNP_SS_(Cas9/sgRPS19) (1.5 mg of Cas9 equiv./kg). Each value was determined from a single deep-sequencing library prepared from genomic DNA. **(C)** Sequence alignment of top‑20 potential off‑target sites uncovered by Site‑seq assay. **(D)** Editing efficiencies at on‑target RPS19 and top‑20 off‑target sites comparing saline and SNP_SS_(Cas9/sgRPS19) treatment groups.

**Supplementary Figure 20. The safety evaluation of SNP_SS_(Cas9/sgRPS19) in vivo.**

**(A)** Histological analyses of major organs excised from healthy mice received SNP_SS_(Cas9/sgRPS19) or normal saline. **(B)** Blood analysis of ALB, AST, ALT and ALP from healthy mice intravenous injected of SNP_SS_(Cas9/sgRPS19) or normal saline. **(C)** Body weight and food intake monitoring of healthy mice after treatment with SNP_SS_(Cas9/sgRPS19) or normal saline. **(D)** Survival rates of mice treated with SNP_SS_(Cas9/sgRPS19) versus normal saline. ALB: Albumin; AST: Aspartate Aminotransferase; ALT: Alanine Transaminase; ALP: Alkaline phosphatase. *P* values were determined by two-tailed unpaired t test **(B)** and two-way ANOVA **(C)**. Data are presented as the means ± SD (ns: no significance; **P* < 0.05).
